# Characterizing 5-hydroxymethylcytosine in human prefrontal cortex at single base resolution

**DOI:** 10.1186/s12864-015-1875-8

**Published:** 2015-09-03

**Authors:** Jeffrey A. Gross, Alain Pacis, Gary G. Chen, Luis B. Barreiro, Carl Ernst, Gustavo Turecki

**Affiliations:** McGill Group for Suicide Studies, Douglas Mental Health University Institute, 6875 boul. Lasalle, Montreal, Quebec Canada; Department of Genetics, CHU Sainte-Justine Research Centre, 3175 Chemin de la Côte-Sainte-Catherine, Montreal, Quebec Canada; Departments of Biochemistry and Pediatrics, University of Montreal, 2900 Boulevard Edouard-Montpetit, Montreal, Quebec Canada

**Keywords:** 5-hydroxymethylcytosine, Prefrontal cortex, Human, Epigenetics, Brain

## Abstract

**Background:**

The recent discovery that methylated cytosines are converted to 5-hydroxymethylated cytosines (5hmC) by the family of ten-eleven translocation enzymes has sparked significant interest on the genomic location, the abundance in different tissues, the putative functions, and the stability of this epigenetic mark. 5hmC plays a key role in the brain, where it is particularly abundant and dynamic during development.

**Results:**

Here, we comprehensively characterize 5hmC in the prefrontal cortices of 24 subjects. We show that, although there is inter-individual variability in 5hmC content among unrelated individuals, approximately 8 % of all CpGs on autosomal chromosomes contain 5hmC, while sex chromosomes contain far less. Our data also provide evidence suggesting that 5hmC has transcriptional regulatory properties, as the density of 5hmC was highest in enhancer regions and within exons. Furthermore, we link increased 5hmC density to histone modification binding sites, to the gene bodies of actively transcribed genes, and to exon-intron boundaries. Finally, we provide several genomic regions of interest that contain gender-specific 5hmC.

**Conclusions:**

Collectively, these results present an important reference for the growing number of studies that are interested in the investigation of the role of 5hmC in brain and mental disorders.

**Electronic supplementary material:**

The online version of this article (doi:10.1186/s12864-015-1875-8) contains supplementary material, which is available to authorized users.

## Background

In mammalian cells, 5-methylcytosine (5mC) has been the most widely studied epigenetic mark, and has long been regarded as a stable DNA modification. Recently, however, research has shown that 5mC can be oxidized to 5-hydroxymethylcytosine (5hmC) by the ten-eleven translocation (TET) family of proteins [1–3]. 5hmC has been shown to be most abundant in brain tissue [4–6] and to influence transcriptional regulatory activity [7–10].

Although its role is not yet completely understood, 5hmC has been increasingly investigated in neuropsychiatric phenotypes, likely due to its involvement in neuronal development and enrichment in brain tissue [11, 12]. In humans, global levels of 5hmC have been shown to be reduced in Alzheimer’s disease [13], while site-specific differences have been demonstrated in autism spectrum disorder [14], Huntington’s disease [15], and psychosis [16]. In mouse models, genome-wide alterations have also been observed in Huntington’s disease [17], as well as in fragile X-associated tremor/ataxia syndrome [18]. Taken together, these observations suggest a significant role of 5hmC in the etiology of neuropsychiatric diseases.

To date, however, investigations of 5hmC in the human brain have either been in very small samples or have utilized low-throughput techniques. Here, we use a sample with good power and sequencing resolution to provide insight into genomic characteristics of 5hmC in the brain. Although we observe sizable variability of 5hmC between subjects, lending to the hypothesis of a dynamic cytosine-modifying pathway, we also demonstrate a strong association between stable 5hmC and epigenetic properties. In its entirety, the descriptive data presented here provide a map of 5hmC at single base resolution, which is of interest to future studies of 5hmC in the cortex of the human brain.

## Results

### Sequencing characteristics

**Fig. 1:**
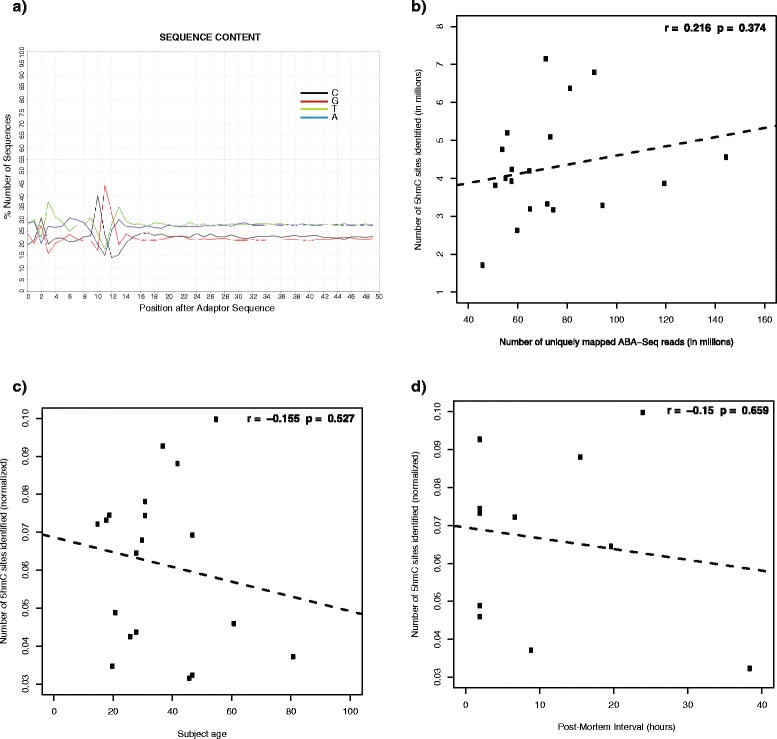
Quality metrics show the efficacy of the AbaSI enzyme, sufficient depth of sequencing, and suitability of sample. **a** The AbaSI enzyme is said to cut 11-13 bp downstream of a glucosylated 5hmC. An example from a sequencing quality control plot showed a peak of cytosine and guanine bases at the 11th and 12th positions of sequencing reads, respectively. **b** No correlation was observed between the number of uniquely mapped AbaSI-Seq reads and the number of 5hmC detected per subject, thereby showing sufficient depth of sequencing. **c**-**d** No correlation was observed between either the subjects’ ages (c) or their PMIs (d) and the total number of 5hmC sites detected

Several high-throughput epigenetic sequencing protocols rely on restriction enzymes, such as MspI and HpaII. In this study, we used AbaSI, an enzyme that recognized glucosyl-modified cytosines and created a cut site 11–13 base pairs downstream of this modified cytosine [19], followed by sequencing to conduct a whole-genome 5hmC analysis at single-base resolution. We initially studied DNA from prefrontal cortical samples from 19 male subjects. As expected, our sequencing results consistently showed that 40–45 % of all reads contained cytosine and guanine bases, respectively, at the 11th and 12th positions after the adaptor sequence (Fig. 1a). This confirmed the specificity of the enzyme, as 5hmC occurs almost exclusively in CpG sequences in both fetal and adult mammalian brain [8, 9]. This value was also expected since, methodologically, half of the sequenced reads were not expected to contain a CpG dinucleotide at these positions and, therefore, were discarded during bioinformatic analyses. We generated an average of 109,394,675 single end reads per subject, of which an average of 82,210,131 ± 4,498,459 reads passed multiple filtering steps and were used to generate the data presented here (Additional file 1). We plotted the number of aligned reads against the number of unique 5hmC sites detected and found that 60–80 million reads was an optimal range; there were four subjects with >90 million uniquely aligned reads and the number of 5hmC sites in these subjects was not notably different from most subjects with 60–80 million reads (Fig. 1b). This sugested diminishing returns of 5hmC detectabiliy under these experimental parameters after ~60–80 million reads. We next assessed the effects of age and post-mortem delay, two factors that might affect 5hmC leves, and found no signficant correlation (Fig. 1c, d).

### Genome-wide inter-individual differences of 5hmC

A major focus of this study was to determine whether 5hmC sites were present at similar genomic locations in brain tissue from different individuals. We first investigated what proportion of all 5hmC sites were common to multiple individuals. We identified a total of 17,368,538 unique 5hmC sites across 19 individuals, of which 81.3 % were also identified in a previous study examining 5hmC content in the prefrontal cortex using Tet-assisted bisulfite sequencing (TAB-Seq) [9]. We found that 41.7 % of all 5hmC sites observed were found in at least 5 individuals, whereas 12.2 % of all 5hmC sites were common to at least 10 individuals, and only 1.3 % were common to at least 15 individuals (Additional file 2). As a point of comparison, we investigated the proportion of 5mC sites common to multiple individuals by whole genome bisulfite sequencing (WGBS). 5mC showed considerable stability across the 19 subjects investigated when considering 5, 10, or 15 individuals, however we observed an abrupt decrease in the percentage of sites common to all 19 subjects (Additional file 3). Although these data indicated that 5hmC is considerably more variable than 5mC, they also suggested a relative degree of stability of 5hmC sites in brain tissue from different individuals.

We next assessed the probability of detecting similar 5hmC sites between any two individuals. To do so, we selected two representative samples with respect to sequencing depth and number of unique 5hmC sites. We subsequently calculated the hypergeometric probability of detecting the same 5hmCs in sequencing reads across independent samples. First, we estimated the total number of 5hmC sites in the human brain genome to be around 27 million, based on previously published data [9]. We observed 6,793,582 and 3,818,749 total 5hmC sites in each of the representative samples considered in this analysis, of which 1,999,493 5hmC sites were common to both subjects (*P* < 3.34e^−178^), suggesting that the pattern of common 5hmC sites observed between samples was not random.

### Chromsomal distribution of 5hmC in human prefrontal cortex

**Fig. 2:**
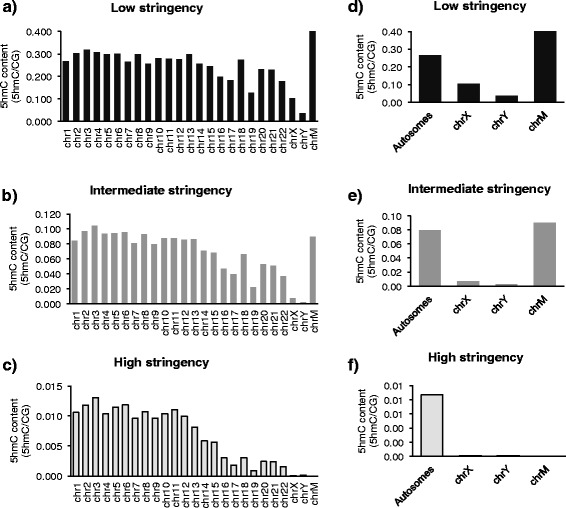
The total number of hydroxymethylated CGs per chromosome decreased exponentially with increasing stringency. Plots displaying the total number of 5hmC sites for each chromosome corrected for the total number of CGs on the respective chromosome, for low (**a**), intermediate (**b**), and high (**c**) stringencies. Summary plots for autosomes, sex chromosomes, and mitochondrial DNA are shown for each respective stringency (**d**-**f**)

While we observed a strong overlap of 5hmC across individuals, we opted to perform the following analyses according to three levels of stringency; low, intermediate, and high stringencies, which represented all 5hmC sites common to ~25, 50, and 75 % of the total sample, respectively. In the low stringency group, an average of 25.7 ± 1.1 % of all autosomal CGs contained a hydroxymethyl mark, while 7.4 ± 2.3 % and 0.8 ± 0.4 % of CGs contained a hydroxymethyl mark in the intermediate and high stringency groups, respectively (Fig. 2a–f, Additional file 4).

The total number of 5hmC sites analyzed in the intermediate stringency model was 2,121,951, which was slightly lower than the average number of 5hmC sites observed in any single individual (4,277,308 ± 319,846). Furthermore, 94.75 % of sites in the intermediate stringency were also present in a recent TAB-Seq study of the prefrontal cortex of one individual human female [9]. For these reasons, we focused further analyses on the intermediate stringency model.

### 5hmC densities across chromosomal features

**Fig. 3:**
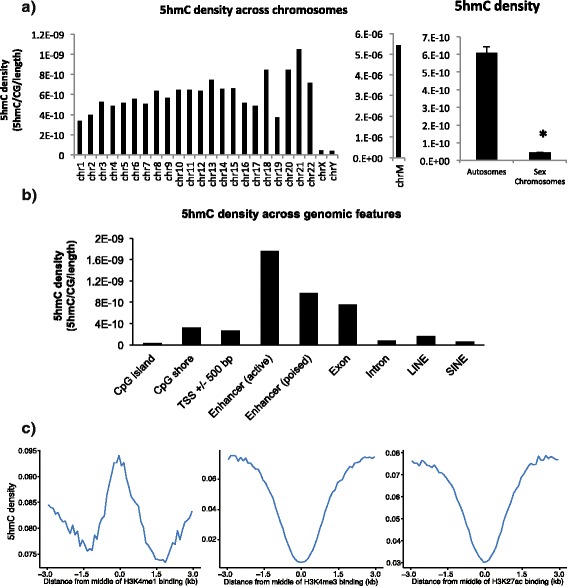
5hmC densities on chromosomes, within genomic features, and around histone modification binding sites. **a** 5hmC density remained relatively stable from one chromosome to the next, although the density appeared to show more variability on shorter chromosomes. 5hmC density was also far greater on autosomal chromosomes than the two sex chromosomes (two-tailed *P* < 0.0001). **b** 5hmC was most highly abundant in enhancer elements and gene bodies, while its density decreased in CpG islands, the TSS, and repetitive elements. **c** Increased 5hmC was observed at the H3K4me1 binding site, while the opposite was true for both H3K4me3 and H3K27ac binding sites

We next determined the density of 5hmC on each chromsome and within specific chromosomal features. We defined the density as being the total number of 5hmC sites corrected for the total number of CGs on the chromosome and the chromosome length. We observed the density of 5hmC to be quite stable across autosomal chromosomes, although a relative increase in density was observed in chromosomes 18, 20, 21, and 22 compared to other chromosomes. Interestingly, in comparison to the autosomes, the density of 5hmC was greatly decreased in the sex chromosomes and increased in mitochondrial DNA (Fig. 3a).

The density of 5hmC across chromosomal features is of interest as it may provide insight into its potential functional properties. Genomic features were defined based on GRCh37/hg19 genomic annotations downloaded from the UCSC browser and Ensembl release 75. Our data showed an increase in 5hmC density in both poised and active enhancers, as well as in exons (Fig. 3b). The increase in 5hmC in these regions has been previously observed in human embryonic stem cells [20, 21] and both fetal and adult mouse brain [8]. In contrast, 5hmC density appeared to be relatively depleted in transcription start sites (TSS), CpG shores, and, most strikingly, CpG islands and both long and short interspersed elements (LINEs and SINEs, respectively).

### Potential functional properties of 5hmC

The observed increase in 5hmC density in enhancer regions of the genome led to the hypothesis that 5hmC may also be associated with histone marks on an individual basis. Using data from chromatin maps in the frontal lobe [22], we calculated the mean 5hmC levels in contiguous 100 bp bins flanking the proposed histone modification binding site. Consistent with the results showing increased density of 5hmC in poised enhancer regions (Fig. 3b), we also observed an increase in 5hmC density at the H3K4me1 ChIP-Seq peak (Fig. 3c). In addition, opposite results were seen at both the H3K4me3 and H3K27ac ChIP-Seq peaks, as the density of 5hmC decreased at the midpoint for both histone marks (Fig. 3c).

**Fig. 4:**
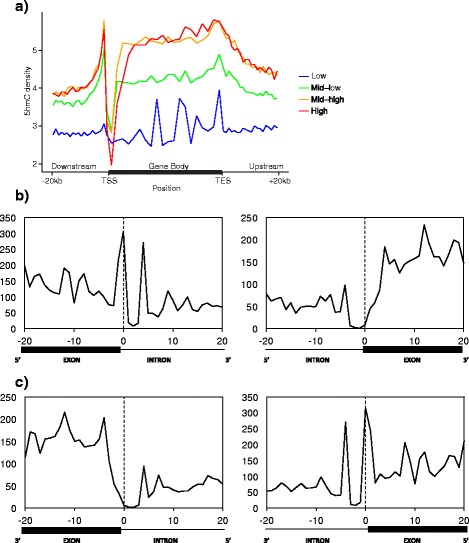
5hmC was enriched in the gene bodies of highly expressed genes and at exon-intron boundaries. **a** 5hmC density was plotted against genomic location for all genes separated in quartiles by their expression levels. 5hmC showed the greatest decrease in density at the TSS for highly expressed genes, while also showing the greatest increase in density in the gene body for this same group. The opposite trend was seen in genes in the lowest quartile of expression. An interesting spike in 5hmC density was also observed at the TES in all quartiles of expression. **b** 5hmC density is increased in the exons on the 5’ and 3’ ends of exon-intron boundaries. **c** A similar pattern of 5hmC density was also seen on the anti-sense strand of corresponding exon-intron boundaries. (TSS = transcription start site; TES = transcription end site)

Similar to histone and cytosine methylation, 5hmC may be an epigenetic mark that associates with altered gene transcription. To explore this possibility further, we compared 5hmC densities across the gene body to available RNA-Seq data from the prefrontal cortex of 11 independent control samples [23]. We plotted 5hmC density along the gene body for all genes separated in quartiles by their expression levels. We found that 5hmC was enriched in the gene bodies of highly expressed genes (Fig. 4a). This was consistent with similar associations between gene body 5hmC and actively transcribed genes reported previously in limited human and mouse brain samples [8, 9], as well as mouse neuronal cells [7]. In addition to gene body 5hmC, genes with the highest expression also showed the greatest reduction in 5hmC at the TSS (Fig. 4a), even though the TSS of highly expressed genes had an overall increase in CpG density compared to the TSS of lowly expressed genes.

Along with the putative function of 5hmC in regulating gene transcription, two previous reports have also suggested an association between 5hmC and exon-intron boundaries [9, 24]. We looked at 5hmC content across 20 bp flanking the 5’ and 3’ ends of 144,157 internal exons spanning 20,745 genes. We observed patterns of increased 5hmC at exon-intron boundaries, specifically at the 5’ splice site and four bases downstream (Fig. 4b). This spike in 5hmC was not strand-specific, as similar results were obtained on both the sense and anti-sense strands (Fig. 4c). The present results may be indicative of a potential splicing mechanism, perhaps through the association of 5hmC and the CCCTC-binding factor (CTCF). Not only is CTCF a transcription factor that has been linked to alternative splicing by regulating the activity of RNA polymerase II [25], but it has also been shown to interact with 5hmC and TET enzymes in both mouse and human embryonic stem cells [21, 26, 27]. Taken together, these results provide an interesting avenue for further research into alternative splicing mechanisms.

### Brain 5hmC clusters are predominant in genes associated with neurodevelopment

Given the association of 5hmC with regulatory processes, we next wanted to determine whether 5hmC aggregated in specific genes. We defined a cluster as being a region containing at least three 5hmCs, each within 200 bp of each other. Using the 2,121,951 5hmC sites in the intermediate stringency category, we identified a total of 65,898 clusters. The midpoint of each cluster was then associated to the TSS of the nearest gene. A total of 8,713 genes were analyzed using GeneTrail [28] for gene ontology (GO). A variety of terms related to cellular, metabolic, and signaling processes were significantly associated with 5hmC clusters. Furthermore, of particular interest, we observed an enrichment of terms associated with neurological processes and neurodevelopment, including terms related to nervous system development, neurogenesis, and gliogenesis (Table 1). Additional significant terms included those related to other areas of development and epigenetic processes. The complete list of GO terms can be found in Additional file 5.

**Table 1:** GO terms related to neurological, epigenetic, and developmental processes from cluster analysis in intermediate stringency

Neurological	Expected	Observed	p-value (fdr)	Enrichment
Neurological system process	616.197	485	2.86E-13	down
Neurogenesis	332.402	396	7.11E-06	up
Neuron projection	190.242	237	1.74E-05	up
Generation of neurons	309.405	366	4.80E-05	up
Neuron differentiation	281.183	333	0.000109238	up
Axon	91.4627	117	0.00142957	up
Cell morphogenesis involved in neuron differentiation	124.389	151	0.00563766	up
Neuron projection morphogenesis	127.525	154	0.00684608	up
Glial cell differentiation	39.721	53	0.0202685	up
Regulation of neuron differentiation	93.0307	113	0.0215321	up
Gliogenesis	46.5153	60	0.0317942	up
Regulation of neurogenesis	113.414	134	0.0340942	up
Epigenetic	Expected	Observed	p-value (fdr)	Enrichment
Protein amino acid methylation	25.0869	35	0.0332904	up
Lysine N-methyltransferase activity	18.8152	27	0.0455873	up
Protein-lysine N-methyltransferase activity	18.8152	27	0.0455873	up
Histone-lysine N-methyltransferase activity	18.8152	27	0.0455873	up
N-methyltransferase activity	29.7907	40	0.0461752	up
Histone H2A acetylation	6.27173	11	0.0496797	up
Development	Expected	Observed	p-value (fdr)	Enrichment
Sensory perception of smell	189.197	33	1.42E-66	down
Sensory perception of chemical stimulus	209.58	50	3.33E-61	down
Sensory perception	389.37	253	6.42E-22	down
Developmental process	1748.24	1912	1.16E-08	up
System development	1318.63	1463	2.08E-08	up
Anatomical structure development	1460.79	1608	4.39E-08	up
Multicellular organismal development	1593.54	1744	6.81E-08	up
Nervous system development	603.131	699	1.78E-07	up
Regulation of developmental process	413.934	494	1.94E-07	up
Positive regulation of developmental process	182.925	236	3.04E-07	up
Neuron development	205.399	249	0.000157004	up
Forebrain development	90.4174	118	0.000419088	up
Central nervous system development	239.371	282	0.000802517	up
Neuron projection development	166.723	200	0.00211809	up
Sensory organ development	129.093	154	0.0128753	up
Regulation of nervous system development	123.344	146	0.0233886	up
Brain development	163.588	189	0.0279154	up
Post-embryonic development	33.4492	45	0.0307565	up
Regulation of neuron projection development	56.4456	71	0.0355767	up
Embryonic development	321.949	354	0.0476929	up

### Sex differences in 5hmC in the human prefrontal cortex

**Fig. 5:**
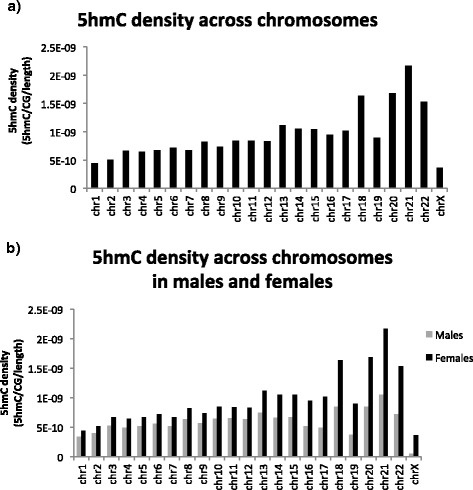
5hmC density in females showed similar patterns as those in males. **a** Similar to males, 5hmC density remained stable across chromosomes, with slight increases observed on chromosomes 18, 20, 21, and 22. **b** 5hmC density between males and females showed similar trends from one chromosome to the next, although gender differences in 5hmC density became more pronounced as the chromosome size decreased

In males, the density of 5hmC on the sex chromosomes (mean = 4.43e^−11^ ± 2.37e^−12^) was nearly 10-fold lower (two-tailed *P* < 0.0001) compared to the autosomes (mean = 6.08e^−10^ ± 3.56e^−11^) (Fig. 3a), suggesting differential 5hmC patterns between chromosome types. To further explore this finding, we performed another AbaSI-Seq analysis using DNA from the prefrontal cortex from females (*n* = 5). Similar to our experiment in males, we used all sites common to a proportion of subjects (at least 3 individuals), which is equivalent to the intermediate stringency model, to plot the density of 5hmC across all chromosomes. As in the analysis with males only, the density of 5hmC was increased on chromosomes 18, 20, 21, and 22, while remaining quite stable across all other autosomes (Fig. 5a). Interestingly, the difference in 5hmC density between males and females became larger as the chromosome size decreased (Fig. 5b).

**Fig. 6:**
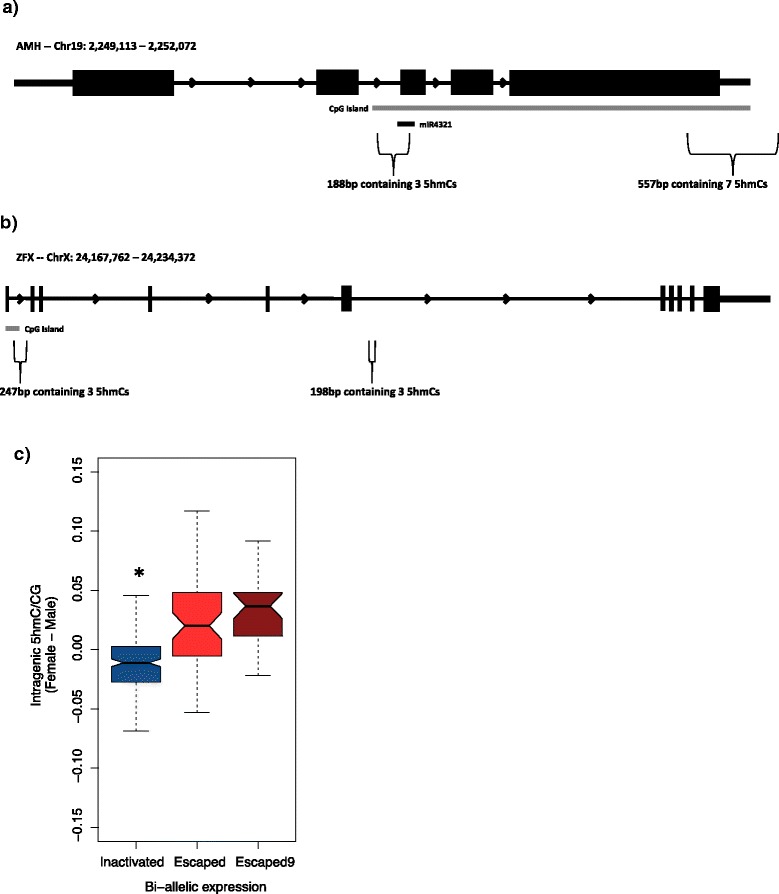
Cluster analysis of 5hmC sites unique to females showed clusters related to gender differentiation. **a** Two 5hmC clusters unique to females were found in the gene body and 3’ UTR of the AMH gene. Both were present within a CpG island, with one also being present around a miRNA binding site. **b** 5hmC clusters unique to females were also present in the ZFX gene that was differentially expressed in females compared to males (log_2_ FC = 0.6533; FDR *p* < 0.0001). **c** Analysis of 5hmC density in females also revealed a significant increase in intergenic 5hmC in genes escaping X-inactivation. Genes most likely to escape (Escaped9) showed a marginal increase in intergenic 5hmC compared to other levels of potential to escape

To identify differences in 5hmC patterns between males and females, we reasoned that differential clusters of 5hmC on any chromosome might indicate potential regulatory regions. As such, to identify 5hmC clusters unique to males, we sampled five randomly selected sets of 5 males and compared 5hmC sites in each set to those found in the females. We identified 27,410 5hmC clusters, associated with 7,064 genes, which were both unique to males and common in all five randomly selected sets. Subsequent GO analysis identified an enrichment in genes related to anatomical and system development, among others (Additional file 6). To determine female-specific 5hmC clusters, we compared all 5hmC sites on autosomes and the sex chromosomes, found in both the female (11,054,815 sites) and male samples (17,368,538 5hmC sites). In doing so, we identified 707,549 5hmC sites unique to the females included in this study, which might represent gender-specific 5hmC sites. A cluster analysis of these 5hmC sites specific to females indicated a total of 12,034 clusters, which were associated to 4,417 genes. GO analysis identified terms related to organ morphogenesis, anatomical structure development, and system development, among others (Table 2, Additional file 7). Of particular interest were 2 clusters on the anti-Müllerian Hormone (AMH) gene, one of which contained three 5hmC sites within a 188 bp region spanning intron 2 and exon 3 and the other containing seven 5hmC sites within a 557 bp region of the 3’ untranslated region (UTR) (Fig. 6a). According to the RNA-Seq data, AMH was expressed in the frontal cortex. We observed increased 5hmC density in the gene body and a spike in 5hmC density at the transcription end site (TES), both of which were in agreement with the two clusters found in the AMH gene. AMH is associated with gender-specific development as it is expressed in males up to puberty to suppress the development of the fallopian tubes and ovaries [29]. In females, however, AMH is most highly expressed between puberty and menopause, as it recruits primary follicles in the ovaries [29]. Given the hypothesis that 5hmC is associated with active transcription, one would expect few to no 5hmC clusters in the AMH gene in males. Indeed, none of the 65,898 clusters found in the male cluster analysis, using all sites common in at least 10 males, were in the AMH gene.

**Table 2:** GO terms related to sexual differentiation and development in 5hmC clusters unique to females

GO term	Expected	Observed	p-value (fdr)	Enrichment
Organ morphogenesis	172.483	249	2.94E-09	up
Anatomical structure morphogenesis	362.983	462	5.47E-08	up
Anatomical structure development	740.538	864	5.41E-07	up
System development	668.472	785	8.92E-07	up
Organ development	502.877	603	2.39E-06	up
Tissue development	215.405	269	0.000475655	up
Anatomical structure formation involved in morphogenesis	121.878	161	0.0010206	up
Regulation of anatomical structure morphogenesis	81.6049	109	0.00786336	up

Using the publicly available RNA-Seq data [23], we investigated genes showing differential expression between genders (Additional file 8). This data showed the X-linked zinc finger protein (ZFX) gene on the X chromosome to be one of the most significantly differentially expressed genes in females (log_2_ FC = 0.653; FDR-corrected *P* < 0.0001). Of the 5hmC clusters unique to females, two are located within the ZFX intergenic region (Fig. 6b). ZFX, which has been shown to escape X-inactivation, is analogous to the Y-linked zinc finger protein (ZFY) on the Y chromosome, and both have been linked to sex determination [30–33]. Similar to AMH, none of the clusters, using all sites common in at least 10 males, were found in the ZFX gene.

### Genes escaping X-inactivation show greater gene body 5hmC density

Following the results showing gender-specific differentially hydroxymethylated regions (DhmRs) in genes related to sexual anatomical development, we hypothesized that 5hmC may play a role in additional gender related developmental processes. 5mC plays an important role in X-inactivation, and previous literature showed that genes escaping X-inactivation have a decrease in promoter CG methylation and an increase in intragenic CH methylation [8]. Previous studies have separated genes on a scale from 1 to 9, with 1 and 9 respectively indicating genes least or most likely to escape inactivation. Using all sites found in our female subjects, our results indicated that these same genes that escape X-inactivation also show a significant increase in gene body 5hmC compared to genes that remain inactivated (Fig. 6c). Furthermore, genes that are most likely to escape X-inactivation showed even greater intergenic 5hmC density. Although this is only an initial characterization, it provides an interesting avenue for future studies investigating the potential etiology of X-linked diseases or disorders.

## Discussion and conclusions

In this study, we provide a deep characterization of the genomic locations of 5hmC in the human prefrontal cortex in a large sample. Using AbaSI-Seq, a high-throughput technique, we confirm previously published work using either low-throughput techniques or single samples. Furthermore, we extend the current knowledge of the role of 5hmC in the brain by linking stable 5hmC sites to enhancer regions and exon-intron junctions, both of which are involved in gene transcription. We also show the existence of regions of the genome that contain gender-specific 5hmC patterns, in addition to providing a putative mechanism for how certain genes escape X-inactivation. These data are of interest, as genomic mapping of 5hmC in the prefrontal cortex will likely be of reference for future studies investigating brain and mental disorders.

Inter-individual variability is common across many fields of study. This is especially true in epigenetics, where both the environment and the genetic landscape are contributing factors in establishing epigenetic features [34–37]. It is, therefore, not surprising to see varying degrees of 5hmC across the chromosomes and specific genomic features. These findings provide a basis for a dynamic DNA demethylation pathway. More importantly, our data show regions of the genome that are consistently hydroxymethylated in many individuals. These results confirm the stability of 5hmC in the human genome and provide a foundation for furthering our understanding of the epigenetic properties of 5hmC. Similarly, we show that, in order to increase our understanding of 5hmC, future research will have to be performed on large samples. Furthermore, increased stringency of bioinformatics analyses will reduce levels of random variability, thereby enhancing the power of studies to infer biological relevance from the 5hmC sequencing data.

In this study, we show that an intermediate level of stringency, defined as 5hmC sites present in at least half the total sample, was appropriate in discovering regions with a consistently high density of 5hmC across subjects. Our results provide reliable evidence that 5hmC is associated with gene regulatory features, such as enhancer regions and histone marks that associate with active gene transcription. Furthermore, we add to the available data in mammalian cells and tissues showing a clear correlation between gene body 5hmC and highly expressed genes. Finally, our cluster analyses show an increased density of 5hmC in genes associated with many developmental processes, specifically those related to neurogenesis and nervous system development. These results are of interest considering the many publications that have linked 5hmC to neurodevelopment, neurodegenerative, and neuropsychiatric phenotypes, and are consistent with the role of 5hmC and 5mC in tissue-specific gene regulation [7, 38], thus supporting the internal validity of our findings. Although the exact function of 5hmC remains unknown, our findings lend to the idea that 5hmC may act as an epigenetic mark by regulating gene transcription.

In addition to characterizing 5hmC in a large sample, we also provide evidence to suggest that 5hmC may be an important factor in anatomical development. Not only do GO analyses of unique DhmRs in females show clusters of 5hmC on genes associated with gender-specific properties and functions, but our results also indicate that genes escaping X-inactivation have increased intragenic 5hmC density. Early research into 5hmC and TET proteins showed specific properties of each based on developmental stages. For example, TET1 and TET2 are more highly expressed in primordial germ cells, while TET3 is primary found in zygotes [39, 40]. Similarly, levels of 5hmC tend to be high in zygotes, decrease rapidly during cell division, and are reestablished in the blastocyst [41]. Interestingly, TET protein knockdown and knockout experiments show that TET proteins appear to have redundant and compensatory roles in establishing 5hmC patterns [40, 42, 43]. As such, although the exact function of 5hmC in development remains unclear, the presence of gender-specific DhmRs highlights the putative roles of 5hmC and TET proteins in embryonic and adult development.

Whether 5hmC is solely an intermediate molecule in the process of DNA demethylation or whether this 6th DNA base may have additional regulatory properties is a question that remains at the forefront of 5hmC research. Recent literature has shown that the oxidation of 5mC to 5hmC occurs on the parental strand after replication, and in the presence of a stable pool of 5hmC in multiple tissues from adult mice [44]. Furthermore, several studies have shown the existence of 5hmC in RNA, from archaeal species to mammalian cells, that occurs through the TET-mediated oxidation of 5mC [45, 46]. Certainly, although 5hmC marks may continue through the oxidation pathway and ultimately lead to a demethylated cytosine, mounting evidence, including those presented here, all suggest that 5hmC does regulate gene transcription. Its mechanism of action, however, remains to be elucidated.

In addition to providing characterizations of 5hmC genomic location and function, we also confirm the efficacy of a high-throughput method to appropriately detect 5hmC sites at single base resolution. AbaSI-Seq, which has been used previously in embryonic stem cells, bases itself on a 5hmC-sensitive restriction enzyme digestion. This technique is valuable as it avoids the biases caused by antibody-based approaches [47] and does not cause DNA damage like other methods that employ bisulfite conversion [48]. Furthermore, AbaSI has independently been shown to recognize both glucosylated and non-glucosylated 5hmC [49], thereby increasing its validity as an effective tool in studying 5hmC.

In summary, this manuscript provides a comprehensive assessment of 5hmC in human prefrontal cortex using a high-throughput technique. Moreover, we provide a reference for comparison of 5hmC in other mammalian species, and shed light on potential avenues of interest for further research in determining the functional relevance of 5hmC.

## Methods

### Subjects

Post-mortem human brain tissue from the prefrontal cortex was obtained from the Douglas – Bell Canada Brain Bank. The prefrontal cortex was chosen given its diverse functions relative to human cognition and of importance to neuropsychiatric phenotypes. Additionally, the prefrontal cortex has been widely implicated in a variety of mental illnesses through dysregulation of epigenetic mechanisms [50–56]. Brain tissue used in this study was dissected at 4 **°**C, snap-frozen in liquid nitrogen, and stored at −80 **°**C following standard procedures. The Quebec Coroner’s office assessed the cause of death for each subject, and subsequently, we obtained information on the subjects’ mental health using psychological autopsies using the Structured Clinical Interviews for DSM-IV Axis 1 [57]. In addition, brain tissue samples from all subjects were assessed for absence of pathological processes by a neuropathologist. Subjects died from either natural (*n* = 11) or accidental (*n* = 13) causes, and all were psychiatrically and neurologically healthy. All subjects were French-Canadian. Mean age was 43.33 ± 4.46 years. Written informed consent was obtained from next-of-kin for all subjects, and the Douglas Institute Research Ethics Board approved this study.

### AbaSI-Seq

Genomic DNA was extracted from the prefrontal cortex using QIAGEN’s QIAamp DNA Mini Kit (QIAGEN, cat. # 51304). Concentrations of genomic DNA were assessed using the Thermo Scientific Nanodrop 1000 spectrophotometer and each sample had a 260/280 ratio greater than 1.8. AbaSI-Seq library construction was performed as described previously [19]. Briefly, DNA was glucosylated by using UDP-glucose and T4-β-glucosyltransferase (New England Biolabs, cat. #M0357L). The DNA was then digested using AbaSI (New England Biolabs, cat. #R0665S) and custom biotinylated adaptors were ligated to DNA ends. The DNA was then sheared using the Covaris S220 Focused-Ultrasonicator (peak incident power = 140; duty % = 10; cycles per burst = 200; time = 120 s). Sheared DNA was captured using Dynabeads® MyOne™ Streptavidin C1 beads (Life Technologies, cat. # 65001). NEBNext™ End Repair Module (New England Biolabs, cat. #E6050L) and NEBNext™ dA-Tailing Module (New England Biolabs, cat. #E6053L) were used to generate blunt ended fragments and create an adenosine tail, respectively. A second set of custom adaptors were ligated to the DNA, followed by PCR amplification using NEBNext™ High-Fidelity 2x PCR Master Mix (New England Biolabs, cat. #E6013AA). Illumina TruSeq indices 1 through 12 were used to barcode samples. 50 cycle, single-read sequencing was performed on Illumina’s HiSeq 2000. A minimum of 60,000,000 sequencing reads was obtained per subject. Sequencing quality control metrics can be found in Additional file 1.

### Data processing

FASTQ reads were filtered based on a quality score greater than 30 and were then aligned to the human reference genome (UCSC Hg19) using Bowtie 2 (http://bowtie-bio.sourceforge.net/bowtie2/index.shtml), with the preset parameters of *very*-*sensitive* (−D 20 -R 3 -N 0 -L 20 -i S,1,0.50). Duplicates were removed using Picard (http://broadinstitute.github.io/picard/) and realignment was performed using the GATK alignment procedures from the Broad Institute (https://www.broadinstitute.org/gatk/). Subsequent filtering of aligned reads was based on a quality score of greater or equal to 10. 5hmC sites were identified using a custom PERL script [19] that detected potential 5hmC sites based on their expected distance from the AbaSI enzymatic cleavage site. A combination of BEDtools, R packages, and custom scripts were used for downstream analyses. Exact specifications are found below.

### Density plots

#### Chromosomes

All 5hmC sites in the intermediate stringency were used to assess chromosomal 5hmC density. Density was defined as the total number of 5hmC sites per chromosome, corrected for the length of the chromosome and the total number of CGs on the chromosome.

#### Genetic features

5hmC sites in the intermediate stringency category were plotted against genomic regions and corrected for the length of the region and the number of CGs within the region. All genomic features were defined based on the GRCh37/hg19 genomic annotation downloaded from the UCSC database. Different genic elements, including transcription start sites (TSS), exons, introns, and transcription end sites (TES), were defined based on the Ensembl (release 75). Since genes can have multiple transcripts, we selected the 5’-most TSS on the positive strand as the single TSS associated with each gene. The reverse (3’ most TSS) was done for genes on the negative strand. We limited downstream analysis to protein-coding genes, resulting in 20,745 TSSs in total. Similarly, annotations for retro-elements (i.e., LINEs and SINEs) and CpG islands were acquired from the UCSC database. CpG shores were defined as the 2 kb flanking a CpG island. Coordinates of predicted of promoter and enhancer regions were obtained from recently published genome-wide maps of chromatin states in the adult brain midfrontal lobe [22], including H3K4me3, H3K4me1 and H3K27ac. Two types of enhancers were distinguished: active enhancers that were simultaneously marked by distal H3K4me1 and H3K27ac, and poised enhancers that were solely marked by distal H3K4me1 [9, 58].

#### ChIP-Seq peaks

To plot 5hmC profiles around ChIP-Seq peaks, the mean 5hmC was calculated for each contiguous 100 bp bin from 3 kb upstream to 3 kb downstream of the central position of the peak.

#### RNA-Seq

Gene expression counts were obtained from RNA-seq data from the preferontal cortex of 11 controls subjects from previously published work [23]. Genes were then classified into quartiles based on their basal gene expression levels: 1st quartile is lowest and 4th is highest. Gene bodies and 20 kb regions upstream and downstream were each divided into 50 intervals. We gathered hydroxymethylation data from windows within each of these intervals and plotted the mean hydroxymethylation level for all windows overlapping each position.

#### Exon-intron boundaries

A total of 144,157 internal exons representing 20,745 genes were retrieved from the Ensembl database, with exclusion of all first and last exons and single-exon genes. 5hmC count was plotted against the 20 bp flanking the 5’ and 3’ exon-intron boundaries on both the sense and anti-sense strands.

### Cluster analyses and gene ontology (GO)

Cluster analyses were performed using online software. Briefly, a region was deemed to have a cluster of 5hmC if there were at least three 5hmCs each within 200 bp of each other. 5hmC clusters was located within a gene body were assigned to that gene, otherwise 5hmC cluster were assigned to the closest TSS from the center position of the 5hmC cluster. GeneTrail [28] was used to test for enrichment of functional annotations among genes nearby 5hmC clusters (<250 kb), using the set of all Ensembl genes as a background. Analysis was done with default parameters and results corrected for multiple testing by the method of Benjamini and Hochberg to control the False Discovery Rate (FDR). GO terms were deemed significant if they had an FDR-corrected *P* ≤ 0.05. The background set included all protein-coding genes.

### Gender analyses

#### Cluster analyses for sites unique to females

5hmC sites in all 5 females were compared to 5hmC sites detected in all 19 males. Cluster analyses, as described above, were performed using only the 5hmC sites unique to females.

#### Cluster analyses for sites unique to males

5 randomly selected sets of 5 males were used to determine male-specific 5hmC clusters. Briefly, all 5hmC sites in each set, separately, were compared to 5hmC sites in all 5 females. Cluster analyses, as described above, were performed in each set using only the 5hmC sites unique to the males in the respective set. Only the clusters that were common in all of the 5 sets of males were deemed to be male-specific.

#### X-inactivation

Genes involved in X-chromosome inactivation were taken from previously published data [59]. Briefly, surveyed genes were given a score from 0–9 based on the number of individual hybrid lines detected from the inactivated X chromosome. A score of 0 corresponds to complete inactivation, whereas a score of 9 corresponds to complete escape from X-inactivation. Intragenic 5hmC patterns on human X-linked genes were then compared between our female and male subjects.

### Availability of supporting data

The data sets supporting the results of this article will be deposited in the GEO database.

## Additional files

Additional file 1:
**is a table containing sequencing quality control metrics.** (PDF 38 kb) Additional file 2:
**is a histogram showing the number of 5hmC sites common across subjects using AbaSI-Seq.** Genomic locations of 5hmC showed high inter-individual variability across all subjects. Stringency categories for downstream analyses were selected based on sample size. Low stringency = sites present in at least 25 % of subjects; intermediate stringency = sites present in at least 50 % of subjects; high stringency = sites present in at least 75 % of subjects. (PDF 40 kb) Additional file 3:
**is a histogram showing the number of 5mC sites common across a different set of subjects using WGBS.** Genomic locations of 5mC showed high degree of inter-individual stability across subjects. Analysis of whole-genome bisulfite sequencing data showed considerable stability in the number of 5mC sites common in 5, 10, and 15 individuals. (PDF 38 kb) Additional file 4:
**shows the percent of CGs on autosomal chromosomes that contain a 5hmC mark.** 5hmC content on autosomal chromosomes showed significant decreases from one stringency to the next. One-way ANOVA (F_(2,65)_ = 372.0; *p* < 0.0001) with Tukey’s Multiple Comparison Test (*p* < 0.05) showed significant decreases in the percent of CGs that can be hydroxymethylated on autosomal chromosomes. Low = Low stringency; Inter = Intermediate stringency; High = High Stringency. (PDF 72 kb) Additional file 5:
**is a table containing GO terms from a cluster analysis using all 5hmC sites in the intermediate stringency.** (PDF 110 kb) Additional file 6:
**is a table containing the GO terms from a cluster analysis using 5hmC sites unique to males.** (PDF 90 kb) Additional file 7:
**is a table containing the GO terms from a cluster analysis using 5hmC sites unique to females. **(PDF 71 kb) Additional file 8:
**is a table containing the results of a differential analysis of gene expression in males and females using previously published RNA-Seq data [** 23 **].** (PDF 76 kb)

## Abbreviations

5hmC: 5-hydroxymethylcytosine
5mC: 5-methylcytosine
TET: Ten-eleven translocation
TAB-Seq: Tet-assisted bisulfite sequencing
WGBS: Whole genome bisulfite sequencing
TSS: Transcription start sites
LINEs: Long interspersed elements
SINEs: Short interspersed elements
CTCF: CCCTC-binding factor
GO: Gene ontology
AMH: Anti-Müllerian Hormone
UTR: Untranslated region
TES: Transcription end site
ZFX: X-linked zinc finger protein
ZFY: Y-linked zinc finger protein
DhmRs: Differentially hydroxymethylated regions

## References

[1] Tahiliani M, Koh KP, Shen Y, Pastor WA, Bandukwala H, Brudno Y (2009). Conversion of 5-methylcytosine to 5-hydroxymethylcytosine in mammalian DNA by MLL partner TET1. Science.

[2] Kriaucionis S, Heintz N (2009). The nuclear DNA base 5-hydroxymethylcytosine is present in Purkinje neurons and the brain. Science.

[3] Ito S, D'Alessio AC, Taranova OV, Hong K, Sowers LC, Zhang Y (2010). Role of Tet proteins in 5mC to 5hmC conversion, ES-cell self-renewal and inner cell mass specification. Nature.

[4] Li W, Liu M (2011). Distribution of 5-hydroxymethylcytosine in different human tissues. J Nucleic Acids.

[5] Kinney SM, Chin HG, Vaisvila R, Bitinaite J, Zheng Y, Esteve PO (2011). Tissue-specific distribution and dynamic changes of 5-hydroxymethylcytosine in mammalian genomes. J Biol Chem.

[6] Nestor CE, Ottaviano R, Reddington J, Sproul D, Reinhardt D, Dunican D (2012). Tissue type is a major modifier of the 5-hydroxymethylcytosine content of human genes. Genome Res.

[7] Mellen M, Ayata P, Dewell S, Kriaucionis S, Heintz N (2012). MeCP2 binds to 5hmC enriched within active genes and accessible chromatin in the nervous system. Cell.

[8] Lister R, Mukamel EA, Nery JR, Urich M, Puddifoot CA, Johnson ND (2013). Global epigenomic reconfiguration during mammalian brain development. Science.

[9] Wen L, Li X, Yan L, Tan Y, Li R, Zhao Y (2014). Whole-genome analysis of 5-hydroxymethylcytosine and 5-methylcytosine at base resolution in the human brain. Genome Biol.

[10] Gabel HW, Kinde B, Stroud H, Gilbert CS, Harmin DA, Kastan NR (2015). Disruption of DNA-methylation-dependent long gene repression in Rett syndrome. Nature.

[11] Szulwach KE, Li X, Li Y, Song CX, Wu H, Dai Q (2011). 5-hmC-mediated epigenetic dynamics during postnatal neurodevelopment and aging. Nat Neurosci.

[12] Hahn MA, Qiu R, Wu X, Li AX, Zhang H, Wang J (2013). Dynamics of 5-hydroxymethylcytosine and chromatin marks in Mammalian neurogenesis. Cell reports.

[13] Coppieters N, Dieriks BV, Lill C, Faull RL, Curtis MA, Dragunow M (2014). Global changes in DNA methylation and hydroxymethylation in Alzheimer's disease human brain. Neurobiol Aging.

[14] Zhubi A, Chen Y, Dong E, Cook EH, Guidotti A, Grayson DR (2014). Increased binding of MeCP2 to the GAD1 and RELN promoters may be mediated by an enrichment of 5-hmC in autism spectrum disorder (ASD) cerebellum. Transcult. Psychiatry.

[15] Villar-Menendez I, Blanch M, Tyebji S, Pereira-Veiga T, Albasanz JL, Martin M (2013). Increased 5-methylcytosine and decreased 5-hydroxymethylcytosine levels are associated with reduced striatal A2AR levels in Huntington's disease. Neruomol Med.

[16] Dong E, Gavin DP, Chen Y, Davis J (2012). Upregulation of TET1 and downregulation of APOBEC3A and APOBEC3C in the parietal cortex of psychotic patients. Transcult. Psychiatry.

[17] Wang F, Yang Y, Lin X, Wang JQ, Wu YS, Xie W (2013). Genome-wide loss of 5-hmC is a novel epigenetic feature of Huntington's disease. Hum Mol Genet.

[18] Yao B, Lin L, Street RC, Zalewski ZA, Galloway JN, Wu H (2014). Genome-wide alteration of 5-hydroxymethylcytosine in a mouse model of fragile X-associated tremor/ataxia syndrome. Hum Mol Genet.

[19] Sun Z, Terragni J, Borgaro JG, Liu Y, Yu L, Guan S (2013). High-resolution enzymatic mapping of genomic 5-hydroxymethylcytosine in mouse embryonic stem cells. Cell reports.

[20] Szulwach KE, Li X, Li Y, Song CX, Han JW, Kim S (2011). Integrating 5-hydroxymethylcytosine into the epigenomic landscape of human embryonic stem cells. PLoS Genet.

[21] Stroud H, Feng S, Morey Kinney S, Pradhan S, Jacobsen SE (2011). 5-Hydroxymethylcytosine is associated with enhancers and gene bodies in human embryonic stem cells. Genome Biol.

[22] Zhu J, Adli M, Zou JY, Verstappen G, Coyne M, Zhang X (2013). Genome-wide chromatin state transitions associated with developmental and environmental cues. Cell.

[23] Akula N, Barb J, Jiang X, Wendland JR, Choi KH, Sen SK (2014). RNA-sequencing of the brain transcriptome implicates dysregulation of neuroplasticity, circadian rhythms and GTPase binding in bipolar disorder. Mol Psychiatry.

[24] Khare T, Pai S, Koncevicius K, Pal M, Kriukiene E, Liutkeviciute Z (2012). 5-hmC in the brain is abundant in synaptic genes and shows differences at the exon-intron boundary. Nat Struct Mol Biol.

[25] Shukla S, Kavak E, Gregory M, Imashimizu M, Shutinoski B, Kashlev M (2011). CTCF-promoted RNA polymerase II pausing links DNA methylation to splicing. Nature.

[26] Feldmann A, Ivanek R, Murr R, Gaidatzis D, Burger L, Schubeler D (2013). Transcription factor occupancy can mediate active turnover of DNA methylation at regulatory regions. PLoS Genet.

[27] Gao F, Xia Y, Wang J, Luo H, Gao Z, Han X (2013). Integrated detection of both 5-mC and 5-hmC by high-throughput tag sequencing technology highlights methylation reprogramming of bivalent genes during cellular differentiation. Epigenetics.

[28] Backes C, Keller A, Kuentzer J, Kneissl B, Comtesse N, Elnakady YA (2007). GeneTrail--advanced gene set enrichment analysis. Nucleic Acids Res.

[29] Zec I, Tislaric-Medenjak D, Megla ZB, Kucak I (2011). Anti-Mullerian hormone: a unique biochemical marker of gonadal development and fertility in humans. Biochemia medica.

[30] Schneider-Gadicke A, Beer-Romero P, Brown LG, Nussbaum R, Page DC (1989). ZFX has a gene structure similar to ZFY, the putative human sex determinant, and escapes X inactivation. Cell.

[31] Palmer MS, Berta P, Sinclair AH, Pym B, Goodfellow PN (1990). Comparison of human ZFY and ZFX transcripts. Proc Natl Acad Sci U S A.

[32] Erickson RP, Zwingman T, Ao A (1993). Gene expression, X-inactivation, and methylation during spermatogenesis: the case of Zfa, Zfx, and Zfy in mice. Mol Reprod Dev.

[33] Mayer A, Lahr G, Swaab DF, Pilgrim C, Reisert I (1998). The Y-chromosomal genes SRY and ZFY are transcribed in adult human brain. Neurogenetics.

[34] Lienert F, Wirbelauer C, Som I, Dean A, Mohn F, Schubeler D (2011). Identification of genetic elements that autonomously determine DNA methylation states. Nat Genet.

[35] McVicker G, van de Geijn B, Degner JF, Cain CE, Banovich NE, Raj A (2013). Identification of genetic variants that affect histone modifications in human cells. Science.

[36] Kasowski M, Kyriazopoulou-Panagiotopoulou S, Grubert F, Zaugg JB, Kundaje A, Liu Y (2013). Extensive variation in chromatin states across humans. Science.

[37] Kilpinen H, Waszak SM, Gschwind AR, Raghav SK, Witwicki RM, Orioli A (2013). Coordinated effects of sequence variation on DNA binding, chromatin structure, and transcription. Science.

[38] Pai AA, Bell JT, Marioni JC, Pritchard JK, Gilad Y (2011). A genome-wide study of DNA methylation patterns and gene expression levels in multiple human and chimpanzee tissues. PLoS Genet.

[39] Hajkova P, Jeffries SJ, Lee C, Miller N, Jackson SP, Surani MA (2010). Genome-wide reprogramming in the mouse germ line entails the base excision repair pathway. Science.

[40] Gu TP, Guo F, Yang H, Wu HP, Xu GF, Liu W (2011). The role of Tet3 DNA dioxygenase in epigenetic reprogramming by oocytes. Nature.

[41] Ruzov A, Tsenkina Y, Serio A, Dudnakova T, Fletcher J, Bai Y (2011). Lineage-specific distribution of high levels of genomic 5-hydroxymethylcytosine in mammalian development. Cell Res.

[42] Dawlaty MM, Breiling A, Le T, Raddatz G, Barrasa MI, Cheng AW (2013). Combined deficiency of Tet1 and Tet2 causes epigenetic abnormalities but is compatible with postnatal development. Dev Cell.

[43] Dawlaty MM, Ganz K, Powell BE, Hu YC, Markoulaki S, Cheng AW (2011). Tet1 is dispensable for maintaining pluripotency and its loss is compatible with embryonic and postnatal development. Cell Stem Cell.

[44] Bachman M, Uribe-Lewis S, Yang X, Williams M, Murrell A, Balasubramanian S (2014). 5-Hydroxymethylcytosine is a predominantly stable DNA modification. Nat Chem.

[45] Fu L, Guerrero CR, Zhong N, Amato NJ, Liu Y, Liu S (2014). Tet-mediated formation of 5-hydroxymethylcytosine in RNA. J Am Chem Soc.

[46] Huber SM, van Delft P, Mendil L, Bachman M, Smollett K, Werner F (2015). Formation and Abundance of 5-Hydroxymethylcytosine in RNA. Chembiochem.

[47] Thomson JP, Hunter JM, Nestor CE, Dunican DS, Terranova R, Moggs JG (2013). Comparative analysis of affinity-based 5-hydroxymethylation enrichment techniques. Nucleic Acids Res.

[48] Grunau C, Clark SJ, Rosenthal A (2001). Bisulfite genomic sequencing: systematic investigation of critical experimental parameters. Nucleic Acids Res.

[49] Horton JR, Borgaro JG, Griggs RM, Quimby A, Guan S, Zhang X (2014). Structure of 5-hydroxymethylcytosine-specific restriction enzyme, AbaSI, in complex with DNA. Nucleic Acids Res.

[50] Cheung I, Shulha HP, Jiang Y, Matevossian A, Wang J, Weng Z (2010). Developmental regulation and individual differences of neuronal H3K4me3 epigenomes in the prefrontal cortex. Proc Natl Acad Sci U S A.

[51] Shulha HP, Cheung I, Whittle C, Wang J, Virgil D, Lin CL (2012). Epigenetic signatures of autism: trimethylated H3K4 landscapes in prefrontal neurons. Arch Gen Psychiatry.

[52] Numata S, Ye T, Hyde TM, Guitart-Navarro X, Tao R, Wininger M (2012). DNA methylation signatures in development and aging of the human prefrontal cortex. Am J Hum Genet.

[53] Gross JA, Fiori LM, Labonte B, Lopez JP, Turecki G (2012). Effects of promoter methylation on increased expression of polyamine biosynthetic genes in suicide. J Psychiatr Res.

[54] Cruceanu C, Alda M, Nagy C, Freemantle E, Rouleau GA, Turecki G (2013). H3K4 tri-methylation in synapsin genes leads to different expression patterns in bipolar disorder and major depression. Int J Neuropsychopharmacol.

[55] Nagy C, Suderman M, Yang J, Szyf M, Mechawar N, Ernst C (2014). Astrocytic abnormalities and global DNA methylation patterns in depression and suicide. Mol Psychiatry.

[56] Lopez JP, Lim R, Cruceanu C, Crapper L, Fasano C, Labonte B (2014). miR-1202 is a primate-specific and brain-enriched microRNA involved in major depression and antidepressant treatment. Nat Med.

[57] Dumais A, Lesage AD, Lalovic A, Seguin M, Tousignant M, Chawky N (2005). Is violent method of suicide a behavioral marker of lifetime aggression?. Am J Psychiatry.

[58] Creyghton MP, Cheng AW, Welstead GG, Kooistra T, Carey BW, Steine EJ (2010). Histone H3K27ac separates active from poised enhancers and predicts developmental state. Proc Natl Acad Sci U S A.

[59] Carrel L, Willard HF (2005). X-inactivation profile reveals extensive variability in X-linked gene expression in females. Nature.

