# Supplementary material for: Characterizing 5-hydroxymethylcytosine in human prefrontal cortex at single base resolution
Source: BMC Genomics. 2015 Sep 3;16(1):672. doi: 10.1186/s12864-015-1875-8 (PMC4559220; doi:10.1186/s12864-015-1875-8)
Supplement: Additional file 1: — is a table containing sequencing quality control metrics. (PDF 38 kb) [file 12864_2015_1875_MOESM1_ESM.pdf]

Supplementary Table S1 - Sequencing quality control metrics

MALES

Subject #	Total Sequencing Reads	Number of Bases from Sequencing	Average Quality Phred Score	Total Reads After Filtering	Unmapped	Uniquely mapped	Mapped >1 time	% mapped	% Uniquely mapped	Total Mapped Reads Used	Total Reads Discarded	% Reads Used
**1**	136,425,815	6,821,290,750	38	126,528,050	2,310,493	91,282,575	32,934,982	98.09%	72.14%	108,529,705	17,998,345	85.78%
**2**	75,824,476	3,791,223,800	37	70,082,708	1,292,962	51,206,900	17,582,846	98.18%	73.07%	60,282,828	9,799,880	86.02%
**3**	109,746,078	5,487,303,900	38	101,002,320	1,462,771	73,548,037	25,991,512	98.56%	72.82%	87,183,867	13,818,453	86.32%
**4**	84,572,609	4,228,630,450	38	78,194,636	1,168,318	57,786,936	19,239,382	98.51%	73.90%	68,099,512	10,095,124	87.09%
**5**	89,383,168	4,469,158,400	37	82,702,245	2,005,032	60,065,746	20,631,467	97.58%	72.63%	70,396,302	12,305,943	85.12%
**6**	121,047,850	6,052,392,500	37	111,988,680	2,323,897	81,512,309	28,152,474	97.93%	72.79%	95,719,919	16,268,761	85.47%
**7**	213,428,878	10,671,443,900	37	197,945,345	4,629,847	144,682,781	48,632,717	97.66%	73.09%	119,981,699	77,963,646	60.61%
**8**	95,119,554	4,755,977,700	38	88,135,532	1,526,884	65,298,422	21,310,226	98.27%	74.09%	76,713,285	11,422,247	87.04%
**9**	111,309,001	5,565,450,050	38	102,956,130	3,588,684	74,631,028	24,736,418	96.52%	72.49%	87,176,540	15,779,590	84.67%
**10**	108,084,545	5,404,227,250	37	99,995,726	2,073,028	71,643,257	26,279,441	97.93%	71.65%	84,810,820	15,184,906	84.81%
**11**	82,324,742	4,116,237,100	37	75,522,284	2,017,406	55,373,629	18,131,249	97.33%	73.32%	64,119,269	11,403,015	84.90%
**12**	80,214,840	4,010,742,000	37	73,915,123	1,450,954	54,029,593	18,434,576	98.13%	73.10%	63,356,251	10,558,872	85.71%
**13**	87,360,096	4,368,004,800	37	78,673,513	1,155,950	57,936,217	19,581,346	98.55%	73.64%	68,306,598	10,366,915	86.82%
**14**	82,368,412	4,118,420,600	38	76,481,727	978,499	56,076,909	19,426,319	98.72%	73.32%	66,345,193	10,136,534	86.75%
**15**	69,657,797	3,482,889,850	38	63,983,839	1,075,204	46,030,200	16,878,435	98.36%	71.94%	54,598,304	9,385,535	85.33%
**16**	144,127,403	7,206,370,150	37	131,907,308	2,825,594	94,634,513	34,447,201	97.86%	71.74%	109,395,311	22,511,997	82.93%
**17**	184,968,494	9,248,424,700	37	164,060,507	2,297,036	119,714,703	42,048,768	98.60%	72.97%	114,568,439	49,492,068	69.83%
**18**	95,793,335	4,789,666,750	38	88,767,704	1,010,211	65,032,196	22,725,297	98.87%	73.26%	77,039,874	11,727,830	86.79%
**19**	106,741,748	5,337,087,400	37	98,307,417	1,140,399	72,325,164	24,841,854	98.84%	73.57%	85,368,784	12,938,633	86.84%
**Mean**	**109,394,675.84**	**5,469,733,792.11**	**37.30**	**100,586,883.89**	**1,912,272.05**	**73,305,848.16**	**25,368,763.68**	**98.13%**	**72.92%**	**82,210,131.58**	**18,376,752.32**	**83.62%**

FEMALES

Subject #	Total Sequencing Reads	Number of Bases from Sequencing	Average Quality Phred Score	Total Reads After Filtering	Unmapped	Uniquely mapped	Mapped >1 time	% mapped	% Uniquely mapped	Total Mapped Reads Used	Total Reads Discarded	% Reads Used
**1**	80,909,825	4,045,491,250	38	74,577,671	1,157,379	54,076,828	19,343,464	98.45%	72.51%	64,226,058	10,351,613	86.12%
**2**	91,406,229	4,570,311,450	38	84,218,190	1,354,303	60,935,424	21,928,463	98.40%	72.35%	72,300,054	11,918,136	85.85%
**3**	102,567,869	5,128,393,450	37	94,703,474	1,127,697	69,445,947	24,129,830	98.81%	73.33%	82,237,690	12,465,784	86.84%
**4**	85,529,099	4,276,454,950	38	78,743,874	1,337,734	57,515,830	19,890,310	98.30%	73.04%	67,964,503	10,779,371	86.31%
**5**	87,133,194	4,356,659,700	38	80,353,815	1,382,176	58,744,264	20,227,375	98.28%	73.11%	69,107,852	11,245,963	86.00%
**Mean**	**89,509,243.20**	**4,475,462,160.00**	**37.80**	**82,519,404.80**	**1,271,857.80**	**60,143,658.60**	**21,103,888.40**	**98.45%**	**72.87%**	**71,167,231.40**	**11,352,173.40**	**86.22%**
