# Supplementary figures and images for: Characterizing 5-hydroxymethylcytosine in human prefrontal cortex at single base resolution
Source: BMC Genomics. 2015 Sep 3;16(1):672. doi: 10.1186/s12864-015-1875-8 (PMC4559220; doi:10.1186/s12864-015-1875-8)

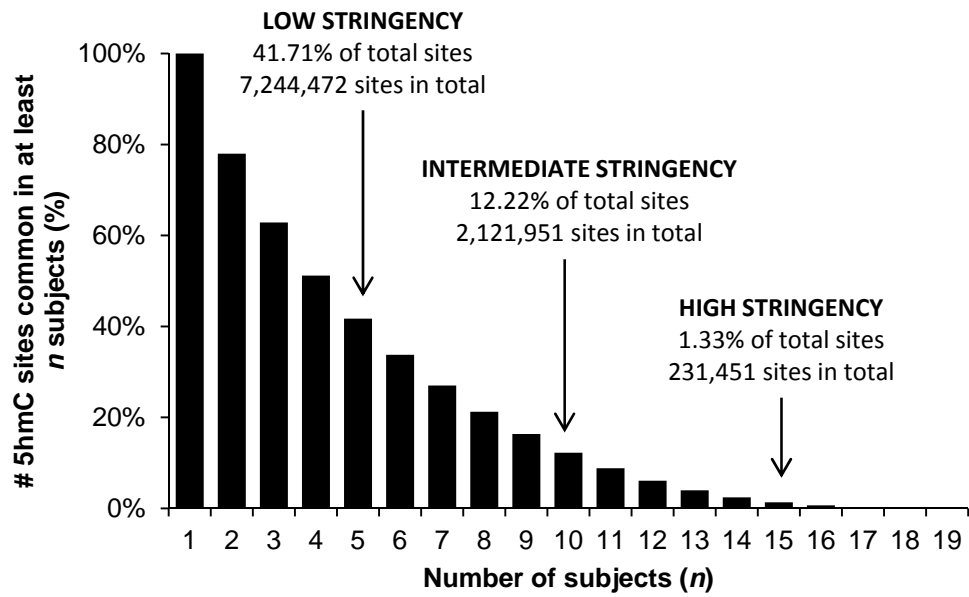

Supplementary figure-S1 (Turecki)

Supplement: Additional file 2: — is a histogram showing the number of 5hmC sites common across subjects using AbaSI-Seq. Genomic locations of 5hmC showed high inter-individual variability across all subjects. Stringency categories for downstream analyses were selected based on sample size. Low stringency = sites present in at least 25 % of subjects; intermediate stringency = sites present in at least 50 % of subjects; high stringency = sites present in at least 75 % of subjects. (PDF 40 kb) [file 12864_2015_1875_MOESM2_ESM.pdf]

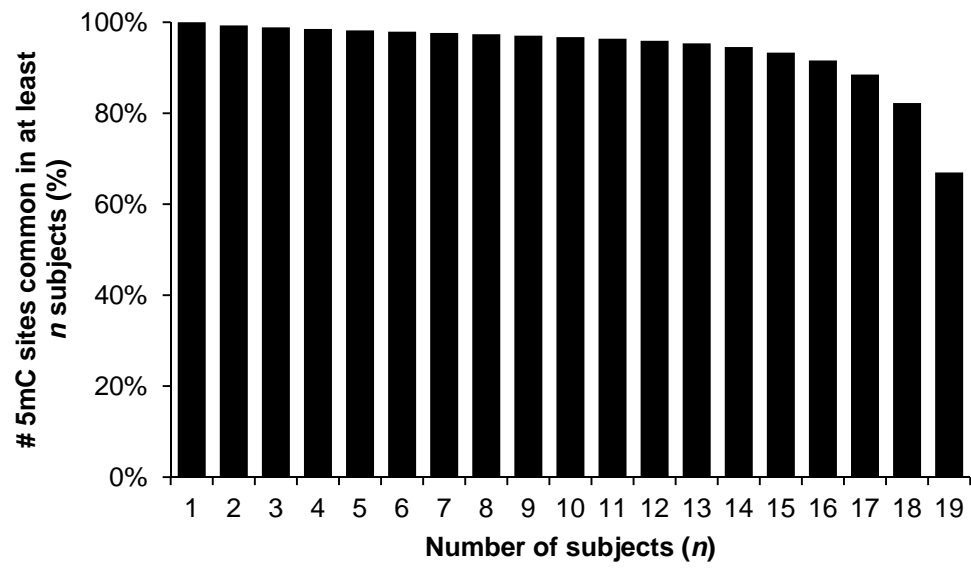

Supplementary figure-S2 (Turecki)

Supplement: Additional file 3: — is a histogram showing the number of 5mC sites common across a different set of subjects using WGBS. Genomic locations of 5mC showed high degree of inter-individual stability across subjects. Analysis of whole-genome bisulfite sequencing data showed considerable stability in the number of 5mC sites common in 5, 10, and 15 individuals. (PDF 38 kb) [file 12864_2015_1875_MOESM3_ESM.pdf]

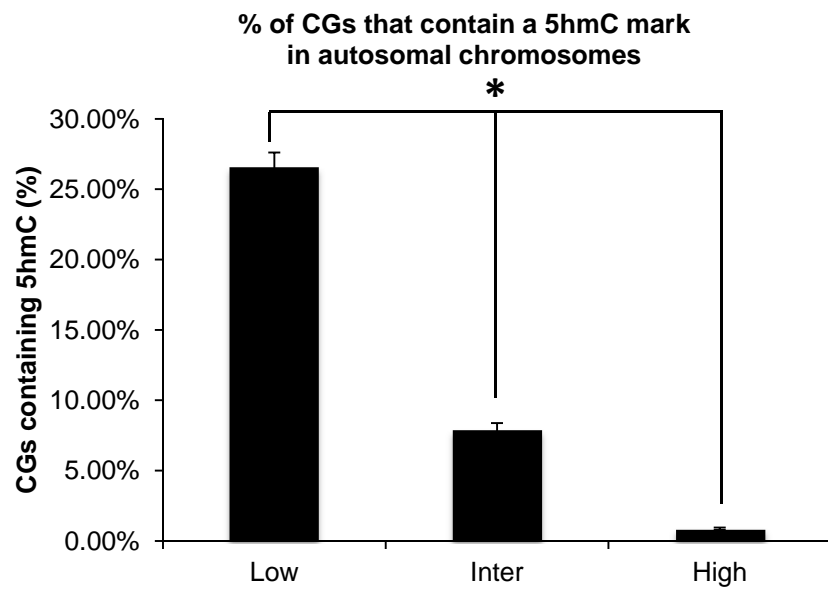

**Supplementary figure-S3 (Turecki)**

Supplement: Additional file 4: — shows the percent of CGs on autosomal chromosomes that contain a 5hmC mark. 5hmC content on autosomal chromosomes showed significant decreases from one stringency to the next. One-way ANOVA (F(2,65) = 372.0; p < 0.0001) with Tukey’s Multiple Comparison Test (p < 0.05) showed significant decreases in the percent of CGs that can be hydroxymethylated on autosomal chromosomes. Low = Low stringency; Inter = Intermediate stringency; High = High Stringency. (PDF 72 kb) [file 12864_2015_1875_MOESM4_ESM.pdf]
