# Supplementary material for: Characterizing 5-hydroxymethylcytosine in human prefrontal cortex at single base resolution
Source: BMC Genomics. 2015 Sep 3;16(1):672. doi: 10.1186/s12864-015-1875-8 (PMC4559220; doi:10.1186/s12864-015-1875-8)
Supplement: Additional file 5: — is a table containing GO terms from a cluster analysis using all 5hmC sites in the intermediate stringency. (PDF 110 kb) [file 12864_2015_1875_MOESM5_ESM.pdf]

Supplementary Table S2 - Cluster analysis in intermediate stringency

GO Terms	expected	observed	p-value (fdr)	enrichment
protein binding	4123.66	4506	1.44E-29	up
cytoplasm	3853.46	4179	1.49E-21	up
binding	6163.54	6451	5.84E-20	up
signaling	1821.94	2063	1.22E-17	up
cellular process	5814.94	6086	1.52E-16	up
protein modification process	899.993	1074	1.56E-16	up
signaling pathway	1302.43	1506	1.82E-16	up
cellular protein metabolic process	1240.23	1438	3.34E-16	up
post-translational protein modification	768.287	928	3.95E-16	up
regulation of signaling pathway	516.895	648	9.85E-16	up
phosphorus metabolic process	658.009	804	1.39E-15	up
phosphate metabolic process	658.009	804	1.39E-15	up
macromolecule modification	941.282	1109	7.44E-15	up
signaling process	1318.63	1511	1.22E-14	up
signal transmission	1315.5	1507	1.50E-14	up
signal transduction	1140.93	1320	2.38E-14	up
positive regulation of biological process	1144.07	1317	2.47E-13	up
phosphorylation	575.431	702	2.66E-13	up
regulation of cell communication	593.724	722	2.77E-13	up
intracellular signaling pathway	777.694	922	4.03E-13	up
protein amino acid phosphorylation	413.411	520	5.84E-13	up
cytoplasmic part	2611.65	2841	8.95E-13	up
protein metabolic process	1473.33	1659	1.56E-12	up
positive regulation of cellular process	1041.63	1202	1.63E-12	up
intracellular	5646.12	5879	4.27E-12	up
intracellular part	5477.31	5708	1.37E-11	up
regulation of biological process	3299.97	3526	4.78E-11	up
death	634.49	755	5.07E-11	up
cell death	632.922	753	5.63E-11	up
regulation of cellular process	3136.39	3356	1.15E-10	up
regulation of signaling process	399.823	495	1.17E-10	up
localization	1699.12	1880	1.17E-10	up
regulation of signal transduction	397.21	492	1.19E-10	up
Golgi apparatus	451.042	551	1.73E-10	up
purine nucleoside binding	814.28	945	2.35E-10	up
biological regulation	3500.67	3721	2.48E-10	up
protein localization	531.006	638	2.61E-10	up
cell communication	858.704	992	2.74E-10	up
negative regulation of biological process	1055.22	1201	2.86E-10	up
programmed cell death	578.044	689	2.92E-10	up
nucleotide binding	1127.87	1277	3.68E-10	up
nucleoside binding	819.506	949	3.75E-10	up
intracellular signal transduction	617.243	730	5.59E-10	up
macromolecule localization	637.103	751	6.86E-10	up
catalytic activity	2604.86	2805	7.75E-10	up
purine nucleotide binding	966.892	1103	1.08E-09	up
apoptosis	573.863	681	1.17E-09	up
negative regulation of cellular process	966.369	1102	1.22E-09	up
adenyl nucleotide binding	795.987	920	1.38E-09	up
ribonucleotide binding	926.125	1056	3.52E-09	up
purine ribonucleotide binding	926.125	1056	3.52E-09	up
adenyl ribonucleotide binding	756.266	874	4.76E-09	up
cellular metabolic process	3712.34	3919	5.23E-09	up
ATP binding	744.245	860	7.00E-09	up
regulation of phosphate metabolic process	282.228	355	8.19E-09	up
regulation of phosphorus metabolic process	282.228	355	8.19E-09	up
establishment of protein localization	458.359	550	9.28E-09	up
regulation of phosphorylation	271.252	342	1.16E-08	up
developmental process	1748.24	1912	1.16E-08	up
system development	1318.63	1463	2.08E-08	up
phosphotransferase activity, alcohol group as acceptor	363.76	444	2.20E-08	up
cellular component organization	1417.41	1565	2.72E-08	up
primary metabolic process	3813.21	4011	3.04E-08	up
Golgi apparatus part	273.866	343	3.23E-08	up
protein transport	450.519	538	3.83E-08	up
Golgi membrane	227.35	290	4.19E-08	up
anatomical structure development	1460.79	1608	4.39E-08	up
establishment of localization	1468.11	1615	5.09E-08	up
transport	1449.81	1595	6.47E-08	up
multicellular organismal development	1593.54	1744	6.81E-08	up
intracellular protein kinase cascade	304.702	375	1.12E-07	up
signal transmission via phosphorylation event	304.702	375	1.12E-07	up
kinase activity	393.551	473	1.13E-07	up
cellular localization	613.062	711	1.13E-07	up
regulation of cell death	460.972	546	1.47E-07	up
regulation of programmed cell death	457.314	542	1.47E-07	up
transferase activity, transferring phosphorus-containing groups	454.7	539	1.55E-07	up
nervous system development	603.131	699	1.78E-07	up
regulation of developmental process	413.934	494	1.94E-07	up
cell surface receptor linked signaling pathway	768.287	875	1.99E-07	up
endomembrane system	735.883	840	2.36E-07	up
positive regulation of developmental process	182.925	236	3.04E-07	up
enzyme linked receptor protein signaling pathway	244.075	305	3.46E-07	up
metabolic process	4182.2	4367	3.62E-07	up

regulation of apoptosis	453.132	535	3.76E-07	up
intracellular membrane-bounded organelle	4179.58	4364	3.80E-07	up
membrane-bounded organelle	4183.24	4367	4.25E-07	up
organelle	4670.35	4852	5.83E-07	up
regulation of small GTPase mediated signal transduction	135.365	180	6.35E-07	up
intracellular organelle	4663.03	4844	6.49E-07	up
transmembrane receptor protein tyrosine kinase signaling pathway	152.089	199	8.22E-07	up
cellular developmental process	985.184	1099	8.40E-07	up
cell proliferation	594.769	684	1.35E-06	up
anatomical structure morphogenesis	716.022	813	1.43E-06	up
positive regulation of cell differentiation	133.797	177	1.45E-06	up
response to organic substance	456.268	534	2.01E-06	up
regulation of transferase activity	208.012	261	2.22E-06	up
protein kinase activity	305.224	369	2.32E-06	up
growth	275.433	336	2.41E-06	up
regulation of kinase activity	200.173	252	2.41E-06	up
cell differentiation	938.669	1046	2.47E-06	up
positive regulation of cell death	242.507	299	2.97E-06	up
positive regulation of metabolic process	538.323	621	3.06E-06	up
positive regulation of programmed cell death	239.894	296	3.06E-06	up
positive regulation of cellular metabolic process	511.669	592	3.49E-06	up
positive regulation of macromolecule metabolic process	499.648	579	3.57E-06	up
regulation of cell proliferation	435.885	510	3.91E-06	up
cell projection	392.506	463	3.91E-06	up
cellular macromolecule metabolic process	2842.14	3001	4.32E-06	up
cellular response to stimulus	558.184	641	4.47E-06	up
establishment of localization in cell	566.024	649	5.05E-06	up
cell development	414.457	486	5.22E-06	up
positive regulation of apoptosis	238.326	293	5.47E-06	up
regulation of protein kinase activity	192.856	242	5.90E-06	up
enzyme binding	326.653	390	6.33E-06	up
neurogenesis	332.402	396	7.11E-06	up
nucleoside-triphosphatase regulator activity	215.852	267	8.78E-06	up
ossification	95.1212	129	1.02E-05	up
regulation of protein metabolic process	332.402	395	1.07E-05	up
intracellular transport	379.44	446	1.12E-05	up
regulation of multicellular organismal process	554.525	634	1.17E-05	up
cellular protein localization	256.096	311	1.19E-05	up
cellular macromolecule localization	257.141	312	1.27E-05	up
response to chemical stimulus	762.015	853	1.52E-05	up
urogenital system development	76.306	106	1.72E-05	up
neuron projection	190.242	237	1.74E-05	up
positive regulation of signaling pathway	197.559	245	1.91E-05	up
positive regulation of nucleobase, nucleoside, nucleotide and nucleic acid metabolic process	349.126	411	2.50E-05	up
regulation of cellular protein metabolic process	294.249	351	2.73E-05	up
regulation of molecular function	547.731	624	2.73E-05	up
kidney development	55.4003	80	3.27E-05	up
MAPKKK cascade	153.135	194	3.36E-05	up
GTPase regulator activity	211.148	259	3.36E-05	up
heart development	125.957	163	3.47E-05	up
transferase activity	856.614	949	3.54E-05	up
positive regulation of transcription	306.792	364	3.54E-05	up
vesicle	365.851	428	3.64E-05	up
positive regulation of nitrogen compound metabolic process	359.579	421	3.94E-05	up
cellular response to organic substance	138.501	177	4.11E-05	up
camera-type eye morphogenesis	27.1775	44	4.12E-05	up
organelle part	2560.96	2701	4.26E-05	up
generation of neurons	309.405	366	4.80E-05	up
positive regulation of kinase activity	126.48	163	4.81E-05	up
organelle organization	753.13	839	4.81E-05	up
cell projection organization	237.28	287	4.82E-05	up
small GTPase mediated signal transduction	221.078	269	5.07E-05	up
macromolecule metabolic process	3130.12	3276	5.27E-05	up
positive regulation of gene expression	323.517	381	5.32E-05	up
regulation of protein amino acid phosphorylation	117.072	152	5.38E-05	up
positive regulation of molecular function	331.879	390	5.41E-05	up
positive regulation of transferase activity	131.184	168	5.81E-05	up
positive regulation of cellular biosynthetic process	382.053	444	5.82E-05	up
negative regulation of cell proliferation	198.082	243	6.30E-05	up
regulation of growth	189.197	233	6.66E-05	up
renal system development	56.9682	81	7.07E-05	up
protein serine/threonine kinase activity	217.42	264	7.64E-05	up
organelle membrane	953.826	1047	7.65E-05	up
cell morphogenesis involved in differentiation	157.316	197	7.80E-05	up
intracellular organelle part	2524.89	2660	7.81E-05	up
positive regulation of cell communication	214.807	261	7.85E-05	up
cell morphogenesis	222.124	269	8.10E-05	up
positive regulation of biosynthetic process	387.802	449	8.46E-05	up
organ development	991.979	1086	8.80E-05	up
neuron differentiation	281.183	333	0.000109238	up
positive regulation of protein kinase activity	121.776	156	0.00012158	up
regulation of Ras protein signal transduction	116.55	150	0.000123982	up
vasculature development	178.744	220	0.00012546	up
cytosol	669.507	747	0.00012546	up
cellular component morphogenesis	244.075	292	0.000130696	up
transcription repressor activity	173.518	214	0.000135712	up

eye morphogenesis	42.8568	63	0.000135712	up
microspike assembly	12.5435	23	0.000137126	up
cytoplasmic vesicle	350.172	407	0.000138365	up
cytoskeletal protein binding	257.141	306	0.000144679	up
blood vessel development	174.563	215	0.000144994	up
small GTPase regulator activity	144.25	181	0.000150051	up
neuron development	205.399	249	0.000157004	up
regulation of ossification	45.47	66	0.000167015	up
regulation of biological quality	871.248	957	0.000170594	up
regulation of cell differentiation	289.022	340	0.000188205	up
actin cytoskeleton organization	142.159	178	0.00021271	up
regulation of anatomical structure morphogenesis	160.974	199	0.000220252	up
filopodium assembly	12.0208	22	0.00023847	up
positive regulation of macromolecule biosynthetic process	360.102	416	0.000242185	up
cell junction	275.956	325	0.000255717	up
Ras protein signal transduction	118.64	151	0.000270148	up
adherens junction	75.2608	101	0.000283557	up
cellular catabolic process	504.352	569	0.000311281	up
bone development	104.006	134	0.000328693	up
regulation of catalytic activity	464.108	526	0.000336536	up
mesenchyme development	42.8568	62	0.000361302	up
cellular response to endogenous stimulus	97.2118	126	0.000374088	up
basolateral plasma membrane	116.55	148	0.000382079	up
actin filament-based process	150.522	186	0.000419088	up
forebrain development	90.4174	118	0.000419088	up
regulation of cellular component organization	279.092	327	0.000419088	up
positive regulation of multicellular organismal process	144.25	179	0.000419088	up
small molecule metabolic process	857.136	938	0.000422953	up
membrane-bounded vesicle	341.287	394	0.000423755	up
regulation of intracellular protein kinase cascade	170.382	208	0.000428307	up
vesicle-mediated transport	342.332	395	0.000438612	up
phosphoinositide binding	58.0135	80	0.000453581	up
cellular component movement	335.015	387	0.000457244	up
negative regulation of cell death	213.239	255	0.000462306	up
positive regulation of signaling process	146.34	181	0.000480749	up
Wnt receptor signaling pathway	97.7344	126	0.00052185	up
negative regulation of cell cycle	97.7344	126	0.00052185	up
blood vessel morphogenesis	151.044	186	0.00054053	up
intracellular protein transport	223.692	266	0.000546542	up
cell projection part	184.493	223	0.000547474	up
nuclear envelope	118.118	149	0.000563336	up
cell migration	236.758	280	0.000598546	up
transcription factor binding	269.162	315	0.000636075	up
cellular response to chemical stimulus	230.486	273	0.000636075	up
negative regulation of macromolecule biosynthetic process	310.973	360	0.000655445	up
positive regulation of RNA metabolic process	266.548	312	0.000670414	up
protein tyrosine kinase activity	72.1249	96	0.0006939	up
protein domain specific binding	199.65	239	0.000713717	up
cell	7430.95	7524	0.000716209	up
guanyl-nucleotide exchange factor activity	77.3513	102	0.000722269	up
polysaccharide biosynthetic process	31.3586	47	0.000736533	up
cellular response to hormone stimulus	93.0307	120	0.000736533	up
cell part	7430.43	7523	0.000772315	up
central nervous system development	239.371	282	0.000802517	up
positive regulation of transcription, DNA-dependent	263.413	308	0.000818452	up
negative regulation of programmed cell death	210.103	250	0.000837817	up
negative regulation of macromolecule metabolic process	422.296	478	0.000853462	up
cytoplasmic membrane-bounded vesicle	331.356	381	0.000853462	up
transmembrane receptor protein kinase activity	42.8568	61	0.000854686	up
ion binding	1894.58	2001	0.000899695	up
regulation of metabolic process	1969.32	2077	0.000923804	up
regulation of protein modification process	193.901	232	0.000923804	up
metal ion binding	1868.45	1974	0.000937764	up
regulation of transcription from RNA polymerase II promoter	388.847	442	0.000951175	up
skeletal system development	198.605	237	0.00097619	up
cation binding	1889.36	1995	0.000976726	up
negative regulation of signaling pathway	139.023	171	0.00112038	up
nuclear part	982.571	1063	0.00112179	up
regulation of erythrocyte differentiation	10.4529	19	0.00113535	up
microtubule cytoskeleton	296.862	343	0.00113913	up
plasma membrane part	1029.09	1111	0.00113913	up
regulation of anatomical structure size	209.058	248	0.00113913	up
positive regulation of signal transduction	143.727	176	0.00123248	up
induction of programmed cell death	170.905	206	0.00124567	up
posttranscriptional regulation of gene expression	124.912	155	0.0012488	up
negative regulation of apoptosis	207.49	246	0.00126981	up
negative regulation of cellular metabolic process	413.411	467	0.00127883	up
focal adhesion	44.9474	63	0.00131541	up
cell-substrate adhesion	77.3513	101	0.00131767	up
negative regulation of metabolic process	454.178	510	0.00133435	up
regulation of Rho protein signal transduction	55.9229	76	0.00136521	up
positive regulation of catalytic activity	285.364	330	0.00137471	up
axon	91.4627	117	0.00142957	up
negative regulation of cellular biosynthetic process	320.903	368	0.00142957	up
negative regulation of biosynthetic process	325.607	373	0.00143553	up
induction of apoptosis	170.382	205	0.00144851	up

negative regulation of cell communication	174.04	209	0.00145	up
cell motility	254.005	296	0.00145	up
localization of cell	254.005	296	0.00145	up
mesenchymal cell differentiation	37.6304	54	0.00145995	up
positive regulation of ossification	14.1114	24	0.00146494	up
peptidyl-amino acid modification	124.389	154	0.00146892	up
transcription cofactor activity	186.061	222	0.00154236	up
positive regulation of MAPKKK cascade	45.9927	64	0.00157098	up
cellular response to stress	350.694	399	0.00178601	up
regulation of MAPKKK cascade	88.3269	113	0.00180042	up
interphase	67.4211	89	0.00180537	up
protein targeting	140.069	171	0.0018069	up
response to inorganic substance	121.253	150	0.00187302	up
transcription from RNA polymerase II promoter	470.902	526	0.00198255	up
activation of protein kinase activity	63.2399	84	0.00198768	up
positive regulation of intracellular protein kinase cascade	116.027	144	0.00202359	up
regulation of cellular metabolic process	1876.29	1976	0.00203585	up
anchoring junction	84.1457	108	0.00204324	up
positive regulation of cell proliferation	234.667	274	0.00208898	up
neuron projection development	166.723	200	0.00211809	up
catabolic process	631.354	694	0.00217741	up
positive regulation of osteoblast differentiation	13.5887	23	0.00235878	up
cell cycle process	342.332	389	0.00241161	up
cell-substrate junction	50.6965	69	0.00247086	up
cellular response to peptide hormone stimulus	50.6965	69	0.00247086	up
cytoskeletal adaptor activity	7.31702	14	0.00247434	up
cell cycle	513.759	570	0.00253478	up
negative regulation of transcription	260.277	301	0.00253478	up
telencephalon development	46.5153	64	0.00253478	up
cellular response to insulin stimulus	46.5153	64	0.00253478	up
interphase of mitotic cell cycle	62.7173	83	0.00253478	up
negative regulation of mitotic cell cycle	11.4982	20	0.00287892	up
camera-type eye development	63.7626	84	0.00294441	up
cell-substrate adherens junction	47.5606	65	0.00307676	up
polypeptide N-acetylgalactosaminyltransferase activity	9.40759	17	0.00311311	up
eye development	80.4872	103	0.00329313	up
site of polarized growth	33.4492	48	0.00330005	up
tube development	175.086	208	0.00331174	up
cellular membrane organization	225.782	263	0.00332597	up
fibroblast growth factor receptor signaling pathway	24.5643	37	0.00335323	up
regulation of osteoblast differentiation	24.5643	37	0.00335323	up
response to endogenous stimulus	260.277	300	0.00346061	up
Ras guanyl-nucleotide exchange factor activity	44.4247	61	0.00379776	up
mesenchymal cell development	36.0624	51	0.00379776	up
endosome transport	36.0624	51	0.00379776	up
membrane organization	226.305	263	0.00401396	up
negative regulation of gene expression	291.635	333	0.00413403	up
negative regulation of tumor necrosis factor production	6.79437	13	0.00437983	up
kinase binding	107.665	133	0.00446537	up
growth cone	32.9266	47	0.00459069	up
response to metal ion	78.3966	100	0.00465437	up
positive regulation of bone mineralization	10.9755	19	0.00469224	up
positive regulation of phosphorus metabolic process	73.1702	94	0.00475824	up
regulation of cell cycle process	73.1702	94	0.00475824	up
positive regulation of phosphate metabolic process	73.1702	94	0.00475824	up
protein oligomerization	99.825	124	0.0049948	up
negative regulation of cell cycle process	22.4737	34	0.0050401	up
positive regulation of anti-apoptosis	17.7699	28	0.00505693	up
membrane	3578.54	3689	0.00515826	up
embryonic camera-type eye development	8.88495	16	0.00517429	up
positive regulation of phosphorylation	71.6022	92	0.00534259	up
transcription activator activity	221.601	257	0.00537773	up
mitotic cell cycle	251.392	289	0.00538542	up
nucleoside triphosphate metabolic process	130.661	158	0.00545415	up
regulation of MAP kinase activity	80.4872	102	0.00559568	up
cell morphogenesis involved in neuron differentiation	124.389	151	0.00563766	up
regulation of cell size	146.34	175	0.00601201	up
nuclear matrix	32.4039	46	0.00625826	up
regulation of Wnt receptor signaling pathway	47.5606	64	0.00627721	up
multicellular organism growth	36.5851	51	0.00634312	up
negative regulation of nucleobase, nucleoside, nucleotide and nucleic acid metabolic process	292.158	332	0.00634312	up
muscle structure development	158.361	188	0.00634312	up
organ growth	25.0869	37	0.0065541	up
ribonucleotide metabolic process	141.114	169	0.00666787	up
response to calcium ion	30.836	44	0.00679627	up
neuron projection morphogenesis	127.525	154	0.00684608	up
cell-matrix adhesion	60.6267	79	0.00686772	up
protein localization in nucleus	79.9645	101	0.00690817	up
negative regulation of nitrogen compound metabolic process	295.294	335	0.00690817	up
nucleoside-triphosphatase activity	367.941	412	0.00696261	up
regulation of multicellular organism growth	29.2681	42	0.00737334	up
positive regulation of multicellular organism growth	10.4529	18	0.00746339	up
protein self-association	6.27173	12	0.0075221	up
positive regulation of erythrocyte differentiation	6.27173	12	0.0075221	up
Rho guanyl-nucleotide exchange factor activity	37.6304	52	0.00766092	up
pyrophosphatase activity	382.575	427	0.00776687	up

ligase activity	220.033	254	0.00808603	up
regulation of cell cycle	233.099	268	0.00813508	up
regulation of cellular component size	175.608	206	0.00824149	up
organ morphogenesis	340.241	382	0.00825252	up
regulation of catabolic process	145.295	173	0.00825252	up
regulation of cell morphogenesis	82.0551	103	0.00825252	up
angiogenesis	120.731	146	0.00836701	up
regulation of cell projection organization	67.9437	87	0.00839655	up
nuclear periphery	36.0624	50	0.00841624	up
membrane-enclosed lumen	971.595	1039	0.00851301	up
lipid biosynthetic process	197.037	229	0.00855956	up
purine ribonucleotide metabolic process	135.365	162	0.00855956	up
proteolysis involved in cellular protein catabolic process	172.995	203	0.00855956	up
respiratory system development	57.4909	75	0.00855956	up
cell leading edge	85.7136	107	0.00859247	up
muscle cell differentiation	74.2155	94	0.00883591	up
glycosaminoglycan biosynthetic process	12.0208	20	0.008918	up
enzyme activator activity	174.04	204	0.008918	up
positive regulation of biomineral formation	12.0208	20	0.008918	up
protein heterooligomerization	30.3134	43	0.00904444	up
dephosphorylation	86.7589	108	0.00932801	up
cell growth	137.455	164	0.00952501	up
peptidyl-tyrosine modification	58.5361	76	0.00958124	up
negative regulation of phosphorus metabolic process	32.9266	46	0.010126	up
cellular macromolecule catabolic process	240.416	275	0.010126	up
regulation of anti-apoptosis	21.4284	32	0.010126	up
negative regulation of phosphate metabolic process	32.9266	46	0.010126	up
Golgi vesicle transport	70.0343	89	0.010126	up
anatomical structure formation involved in morphogenesis	240.416	275	0.010126	up
protein amino acid autophosphorylation	46.5153	62	0.0102595	up
positive regulation of protein amino acid phosphorylation	64.8079	83	0.0104758	up
ER-nucleus signaling pathway	18.2925	28	0.0106813	up
cellular protein catabolic process	174.563	204	0.0106989	up
organelle lumen	953.826	1019	0.0107215	up
cell cycle phase	261.322	297	0.0109587	up
hydrolase activity, acting on acid anhydrides, in phosphorus-containing anhydrides	384.143	427	0.0109899	up
carbohydrate biosynthetic process	71.0796	90	0.01103	up
negative regulation of RNA metabolic process	213.761	246	0.0114354	up
regulation of the force of heart contraction	9.93024	17	0.0114802	up
aging	65.8532	84	0.0114802	up
macromolecule catabolic process	286.932	324	0.0116089	up
regulation of mitotic cell cycle	89.8948	111	0.0116161	up
hydrolase activity	1146.16	1216	0.011669	up
transcription coactivator activity	109.755	133	0.0116916	up
transmembrane receptor protein tyrosine kinase activity	33.9719	47	0.012129	up
regulation of cell development	139.023	165	0.012187	up
protein kinase binding	90.9401	112	0.0125413	up
extracellular matrix	187.107	217	0.0126472	up
polysaccharide metabolic process	64.2852	82	0.0128753	up
sensory organ development	129.093	154	0.0128753	up
heterocycle metabolic process	306.269	344	0.0131317	up
intracellular organelle lumen	935.01	998	0.0135286	up
purine nucleoside triphosphate metabolic process	126.48	151	0.0136154	up
negative regulation of transcription, DNA-dependent	210.626	242	0.0137171	up
regulation of localization	379.44	421	0.013766	up
response to hormone stimulus	233.099	266	0.0138597	up
negative regulation of cytokine production	20.9058	31	0.0140092	up
hydrolase activity, acting on acid anhydrides	386.234	428	0.0141248	up
positive regulation of cellular component organization	116.55	140	0.0141248	up
positive regulation of myeloid cell differentiation	19.3378	29	0.0146556	up
retina development in camera-type eye	19.3378	29	0.0146556	up
regulation of primary metabolic process	1783.78	1865	0.0146556	up
phospholipid binding	96.6892	118	0.0146932	up
negative regulation of phosphorylation	30.836	43	0.0146932	up
peptidyl-tyrosine phosphorylation	57.4909	74	0.0147593	up
nuclear transport	113.936	137	0.0147593	up
regulation of myeloid cell differentiation	37.6304	51	0.0151846	up
nuclear inner membrane	14.634	23	0.0153099	up
peptidyl-threonine modification	14.634	23	0.0153099	up
bone mineralization	25.0869	36	0.0156375	up
osteoblast differentiation	40.2436	54	0.015763	up
Rho protein signal transduction	63.7626	81	0.015763	up
regulation of cellular component movement	125.957	150	0.015763	up
potassium ion transport	70.0343	88	0.0164396	up
axonogenesis	112.368	135	0.0164396	up
positive regulation of transcription from RNA polymerase II promoter	203.831	234	0.0166147	up
tissue morphogenesis	149.999	176	0.0166147	up
muscle tissue development	88.8495	109	0.0166147	up
regulation of transmembrane receptor protein serine/threonine kinase signaling pathway	48.0833	63	0.0166147	up
protein catabolic process	200.173	230	0.0169744	up
transcription regulator activity	764.106	820	0.0172805	up
nucleocytoplasmic transport	113.414	136	0.0173284	up
anti-apoptosis	120.731	144	0.0173703	up
ribonucleoside triphosphate metabolic process	124.389	148	0.0173703	up
response to unfolded protein	31.8813	44	0.0174954	up
insulin receptor signaling pathway	27.7001	39	0.0176082	up

vasculogenesis	21.9511	32	0.0180829	up
muscle organ development	132.752	157	0.0181171	up
interspecies interaction between organisms	172.473	200	0.0181171	up
growth factor binding	56.9682	73	0.0181955	up
regulation of cellular catabolic process	125.435	149	0.0181955	up
biological adhesion	411.321	453	0.018245	up
positive regulation of cell projection organization	34.4945	47	0.0185996	up
protein localization in organelle	108.187	130	0.0190866	up
regulation of biomineral formation	20.3831	30	0.0192705	up
negative regulation of Wnt receptor signaling pathway	30.3134	42	0.0193391	up
cell adhesion	410.798	452	0.020137	up
glial cell differentiation	39.721	53	0.0202685	up
GTPase activator activity	112.891	135	0.0202788	up
purine ribonucleoside triphosphate metabolic process	123.867	147	0.0202788	up
purine nucleotide metabolic process	224.214	255	0.0205068	up
sodium:amino acid symporter activity	5.22644	10	0.0211498	up
cell projection cytoplasm	5.22644	10	0.0211498	up
transforming growth factor beta binding	5.22644	10	0.0211498	up
regulation of cell growth	110.278	132	0.0213612	up
membrane protein proteolysis	17.2473	26	0.0213612	up
microtubule organizing center	140.591	165	0.0215321	up
regulation of neuron differentiation	93.0307	113	0.0215321	up
regulation of nucleotide catabolic process	76.8287	95	0.0216429	up
regulation of purine nucleotide catabolic process	76.8287	95	0.0216429	up
regulation of S phase	12.5435	20	0.0216519	up
acetylgalactosaminyltransferase activity	15.6793	24	0.021903	up
negative regulation of transferase activity	59.0588	75	0.0220556	up
cytoskeleton organization	264.981	298	0.0220636	up
morphogenesis of an epithelium	114.982	137	0.0223765	up
nucleoside phosphate metabolic process	281.183	315	0.0228645	up
nucleotide metabolic process	281.183	315	0.0228645	up
lamellipodium	40.7662	54	0.0228892	up
regulation of nervous system development	123.344	146	0.0233886	up
PDZ domain binding	27.1775	38	0.0233938	up
protein amino acid dephosphorylation	68.989	86	0.024077	up
BMP signaling pathway	33.9719	46	0.02414	up
cell-cell junction	102.438	123	0.0246981	up
perinuclear region of cytoplasm	168.814	195	0.0248027	up
potassium channel activity	66.3758	83	0.0248719	up
regulation of cell adhesion	78.9193	97	0.0249219	up
nuclear import	72.6475	90	0.0250522	up
cell fraction	550.344	596	0.0259935	up
ribonucleotide catabolic process	76.306	94	0.0259935	up
cartilage development	49.6512	64	0.0259935	up
morphogenesis of a branching structure	67.4211	84	0.0270899	up
peptidyl-serine modification	32.4039	44	0.0270899	up
face development	8.88495	15	0.0274617	up
brain development	163.588	189	0.0279154	up
phospholipid biosynthetic process	55.9229	71	0.0279154	up
enzyme regulator activity	436.408	477	0.0279357	up
organelle envelope	331.356	367	0.0279357	up
protein import into nucleus	71.0796	88	0.0281112	up
respiratory tube development	53.3097	68	0.0282616	up
regulation of bone mineralization	18.2925	27	0.0282616	up
regulation of protein localization	90.9401	110	0.0282616	up
striated muscle tissue development	84.6683	103	0.0291355	up
regulation of GTP catabolic process	68.4664	85	0.0291355	up
regulation of GTPase activity	68.4664	85	0.0291355	up
regulation of Ras GTPase activity	59.5814	75	0.029544	up
cofactor binding	124.912	147	0.0296674	up
ureteric bud development	30.836	42	0.029796	up
dorsal/ventral pattern formation	30.836	42	0.029796	up
mRNA metabolic process	188.152	215	0.0301273	up
actin cytoskeleton reorganization	10.4529	17	0.0301273	up
pigment cell differentiation	10.4529	17	0.0301273	up
positive regulation of protein metabolic process	162.02	187	0.0301273	up
morphogenesis of an epithelial fold	10.4529	17	0.0301273	up
negative regulation of transcription from RNA polymerase II promoter	149.999	174	0.0306427	up
transmembrane receptor protein serine/threonine kinase signaling pathway	85.7136	104	0.0306623	up
induction of apoptosis by intracellular signals	26.6548	37	0.0306974	up
post-embryonic development	33.4492	45	0.0307565	up
response to extracellular stimulus	137.978	161	0.0307565	up
GTP catabolic process	69.5117	86	0.0308088	up
envelope	337.628	373	0.0308088	up
regulation of cell morphogenesis involved in differentiation	51.7418	66	0.0310017	up
protein import	93.0307	112	0.0310017	up
lung development	51.7418	66	0.0310017	up
regulation of alpha-beta T cell differentiation	12.0208	19	0.0310017	up
head development	12.0208	19	0.0310017	up
regulation of cartilage development	12.0208	19	0.0310017	up
regulation of response to stress	167.769	193	0.0310017	up
phosphoprotein phosphatase activity	83.1004	101	0.0316422	up
gliogenesis	46.5153	60	0.0317942	up
ubiquitin-dependent protein catabolic process	147.386	171	0.0318289	up
identical protein binding	356.966	393	0.032292	up
phosphoric ester hydrolase activity	173.518	199	0.03251	up

biological_process	7093.85	7164	0.0331136	up
protein modification by small protein conjugation or removal	182.925	209	0.0331136	up
nuclear lumen	768.809	820	0.033185	up
protein amino acid methylation	25.0869	35	0.0332904	up
protein amino acid alkylation	25.0869	35	0.0332904	up
embryonic camera-type eye morphogenesis	6.79437	12	0.0332904	up
cell cycle arrest	61.672	77	0.0338828	up
chromatin modification	166.201	191	0.0338828	up
ubiquitin ligase complex	67.9437	84	0.0340209	up
regulation of neurogenesis	113.414	134	0.0340942	up
foregut morphogenesis	4.7038	9	0.0341654	up
dorsal/ventral axis specification	4.7038	9	0.0341654	up
dendrite cytoplasm	4.7038	9	0.0341654	up
negative regulation of S phase of mitotic cell cycle	4.7038	9	0.0341654	up
lipid metabolic process	464.631	505	0.0343521	up
muscle cell proliferation	34.4945	46	0.0346234	up
melanosome	47.5606	61	0.0348093	up
pigment granule	47.5606	61	0.0348093	up
epithelial to mesenchymal transition	19.3378	28	0.0349969	up
proteinaceous extracellular matrix	158.884	183	0.0349969	up
regulation of nucleotide metabolic process	142.159	165	0.0350973	up
nucleobase, nucleoside and nucleotide metabolic process	292.681	325	0.0351198	up
purine ribonucleotide catabolic process	75.2608	92	0.0355767	up
regulation of neuron projection development	56.4456	71	0.0355767	up
response to nutrient levels	126.48	148	0.0356934	up
regulation of locomotion	126.48	148	0.0356934	up
protein phosphatase binding	23.519	33	0.0361363	up
cellular carbohydrate metabolic process	209.58	237	0.0361363	up
cell division	183.448	209	0.03796	up
nucleoside triphosphate catabolic process	76.306	93	0.0381238	up
cellular polysaccharide metabolic process	32.9266	44	0.0387096	up
response to oxygen levels	79.9645	97	0.0388116	up
positive regulation of cell development	45.9927	59	0.0390843	up
phosphatase binding	26.1322	36	0.039508	up
coenzyme binding	87.2816	105	0.0396758	up
endoplasmic reticulum	518.463	560	0.0399467	up
developmental programmed cell death	8.36231	14	0.0402796	up
positive regulation of lymphocyte differentiation	16.202	24	0.0402796	up
regulation of transport	268.639	299	0.040799	up
energy reserve metabolic process	28.7454	39	0.0416851	up
actin filament organization	71.0796	87	0.0416851	up
small conjugating protein ligase activity	106.619	126	0.0416851	up
positive regulation of protein modification process	122.299	143	0.0417706	up
molecular_function	7631.65	7689	0.0419304	up
activation of MAPKK activity	14.634	22	0.0420179	up
voltage-gated potassium channel activity	49.6512	63	0.0420179	up
mesenchymal cell proliferation	14.634	22	0.0420179	up
SH3 domain binding	49.6512	63	0.0420179	up
regulation of steroid biosynthetic process	14.634	22	0.0420179	up
regulation of morphogenesis of a branching structure	14.634	22	0.0420179	up
positive regulation of stress-activated protein kinase signaling cascade	14.634	22	0.0420179	up
protein dimerization activity	303.134	335	0.0429671	up
steroid biosynthetic process	55.9229	70	0.043011	up
induction of apoptosis by extracellular signals	55.9229	70	0.043011	up
gland morphogenesis	44.4247	57	0.043011	up
clathrin vesicle coat	9.93024	16	0.043011	up
melanocyte differentiation	9.93024	16	0.043011	up
ovulation	9.93024	16	0.043011	up
negative regulation of kinase activity	55.9229	70	0.043011	up
cellular polysaccharide biosynthetic process	20.3831	29	0.043011	up
protein ubiquitination involved in ubiquitin-dependent protein catabolic process	9.93024	16	0.043011	up
aminoglycan biosynthetic process	13.0661	20	0.0431173	up
peptidyl-threonine phosphorylation	13.0661	20	0.0431173	up
nucleoplasm part	304.179	336	0.0431173	up
digestive tract morphogenesis	13.0661	20	0.0431173	up
regulation of cellular response to stress	72.1249	88	0.0434483	up
positive regulation of Wnt receptor signaling pathway	11.4982	18	0.0436227	up
locomotion	315.677	348	0.0436227	up
insoluble fraction	423.864	461	0.0436573	up
negative regulation of protein kinase activity	53.3097	67	0.0436573	up
response to hypoxia	75.7834	92	0.04394	up
protein C-terminus binding	75.7834	92	0.04394	up
nuclear membrane	65.8532	81	0.04394	up
regulation of organelle organization	124.389	145	0.04394	up
regulation of system process	162.542	186	0.04394	up
positive regulation of cellular protein metabolic process	154.18	177	0.0445343	up
nucleobase, nucleoside, nucleotide and nucleic acid catabolic process	105.051	124	0.0445343	up
nucleobase, nucleoside and nucleotide catabolic process	105.051	124	0.0445343	up
peptidyl-serine phosphorylation	27.1775	37	0.0447956	up
protein modification by small protein conjugation	158.884	182	0.0447956	up
cell projection morphogenesis	145.818	168	0.0447956	up
purine nucleotide catabolic process	90.4174	108	0.0450134	up
modification-dependent protein catabolic process	150.522	173	0.0452532	up
modification-dependent macromolecule catabolic process	150.522	173	0.0452532	up
chondrocyte differentiation	18.8152	27	0.0455873	up
ribonucleoside triphosphate catabolic process	73.1702	89	0.0455873	up

purine ribonucleoside triphosphate catabolic process	73.1702	89	0.0455873	up
lysine N-methyltransferase activity	18.8152	27	0.0455873	up
protein-lysine N-methyltransferase activity	18.8152	27	0.0455873	up
histone-lysine N-methyltransferase activity	18.8152	27	0.0455873	up
N-methyltransferase activity	29.7907	40	0.0461752	up
negative regulation of protein amino acid phosphorylation	22.9963	32	0.046192	up
cytoplasmic vesicle membrane	118.118	138	0.0464712	up
dendrite	95.1212	113	0.0468304	up
response to organic nitrogen	40.2436	52	0.047522	up
embryonic development	321.949	354	0.0476929	up
ligase activity, forming carbon-nitrogen bonds	139.546	161	0.0477209	up
cell part morphogenesis	152.612	175	0.0478791	up
purine nucleoside triphosphate catabolic process	74.2155	90	0.0480085	up
regulation of response to external stimulus	96.1665	114	0.0492715	up
response to endoplasmic reticulum stress	17.2473	25	0.0494206	up
labyrinthine layer development	17.2473	25	0.0494206	up
vesicle membrane	123.867	144	0.0494392	up
ovulation from ovarian follicle	6.27173	11	0.0496797	up
clathrin coat of trans-Golgi network vesicle	6.27173	11	0.0496797	up
histone H2A acetylation	6.27173	11	0.0496797	up
olfactory receptor activity	180.312	24	2.90E-71	down
sensory perception of smell	189.197	33	1.42E-66	down
sensory perception of chemical stimulus	209.58	50	3.33E-61	down
G-protein coupled receptor activity	396.164	240	1.11E-28	down
sensory perception	389.37	253	6.42E-22	down
cognition	439.021	309	1.21E-17	down
transmembrane receptor activity	608.88	459	2.77E-17	down
receptor activity	813.234	653	2.73E-15	down
intermediate filament cytoskeleton	91.9854	39	5.09E-14	down
intermediate filament	87.2816	37	2.66E-13	down
neurological system process	616.197	485	2.86E-13	down
signal transducer activity	1037.45	908	3.04E-08	down
molecular transducer activity	1037.45	908	3.04E-08	down
system process	800.168	689	1.12E-07	down
keratin filament	42.8568	19	3.59E-06	down
keratinization	20.9058	5	8.29E-06	down
peptidase inhibitor activity	77.3513	48	3.50E-05	down
endopeptidase inhibitor activity	73.1702	45	4.62E-05	down
endopeptidase regulator activity	74.7381	47	7.72E-05	down
peptidase regulator activity	89.3721	61	0.000270986	down
extracellular region	986.229	899	0.000351576	down
serine-type endopeptidase inhibitor activity	46.5153	27	0.000636075	down
epidermal cell differentiation	42.8568	25	0.00130131	down
ribosome	97.2118	71	0.00166071	down
response to stimulus	1841.28	1745	0.00295325	down
structural constituent of ribosome	78.3966	56	0.00329516	down
serine-type endopeptidase activity	75.7834	54	0.00377863	down
oxygen binding	21.4284	10	0.00503966	down
cytosolic ribosome	38.153	23	0.00516021	down
enzyme inhibitor activity	138.501	112	0.0104862	down
keratinocyte differentiation	38.153	24	0.0104862	down
translational elongation	51.2191	35	0.0112348	down
cysteine-type endopeptidase inhibitor activity	18.8152	9	0.0126909	down
ribosomal subunit	60.1041	43	0.0141016	down
endonuclease activity, active with either ribo- or deoxyribonucleic acids and producing 3'-phosphomonoesters	11.4982	4	0.0163442	down
microtubule-based flagellum	12.5435	5	0.0225522	down
serine hydrolase activity	88.3269	69	0.0238064	down
serine-type peptidase activity	86.7589	68	0.0280638	down
ncRNA processing	97.7344	78	0.0294516	down
nucleosome	27.7001	17	0.029796	down
large ribosomal subunit	31.3586	20	0.0298087	down
cytosolic large ribosomal subunit	18.8152	10	0.030974	down
cytosolic part	75.7834	59	0.037295	down
cell-cell recognition	8.88495	3	0.0416851	down
DNA binding	1145.64	1088	0.0455873	down
rRNA processing	49.1285	36	0.0455873	down
defense response to bacterium	55.9229	42	0.0464737	down
aromatase activity	11.4982	5	0.0474794	down
MHC protein complex	16.7246	9	0.049426	down
