# Supplementary material for: Characterizing 5-hydroxymethylcytosine in human prefrontal cortex at single base resolution
Source: BMC Genomics. 2015 Sep 3;16(1):672. doi: 10.1186/s12864-015-1875-8 (PMC4559220; doi:10.1186/s12864-015-1875-8)
Supplement: Additional file 6: — is a table containing the GO terms from a cluster analysis using 5hmC sites unique to males. (PDF 90 kb) [file 12864_2015_1875_MOESM6_ESM.pdf]

**Supplementary Table S3 - Cluster analysis in 5hmC sites unique to males**

| GO Term | expected | observed | p-value (fdr) | enrichment |
|---|---|---|---|---|
| olfactory receptor activity | 146.187 | 22 | 1.11E-48 | down |
| sensory perception of smell | 153.39 | 31 | 7.22E-44 | down |
| sensory perception of chemical stimulus | 169.916 | 44 | 6.31E-41 | down |
| protein binding | 3343.23 | 3701 | 3.14E-26 | up |
| G-protein coupled receptor activity | 321.187 | 189 | 1.51E-21 | down |
| binding | 4997.05 | 5291 | 1.94E-21 | up |
| biological regulation | 2838.14 | 3152 | 7.53E-21 | up |
| regulation of biological process | 2675.43 | 2977 | 1.22E-19 | up |
| signaling | 1477.12 | 1729 | 2.06E-19 | up |
| regulation of cellular process | 2542.8 | 2836 | 5.23E-19 | up |
| cellular process | 4714.42 | 4981 | 2.30E-16 | up |
| signaling process | 1069.07 | 1269 | 1.24E-15 | up |
| signal transmission | 1066.53 | 1265 | 1.86E-15 | up |
| sensory perception | 315.679 | 209 | 5.00E-14 | down |
| multicellular organismal development | 1291.95 | 1488 | 7.15E-13 | up |
| developmental process | 1417.38 | 1619 | 1.03E-12 | up |
| anatomical structure development | 1184.32 | 1372 | 1.16E-12 | up |
| system development | 1069.07 | 1247 | 2.33E-12 | up |
| signaling pathway | 1055.93 | 1229 | 8.49E-12 | up |
| nervous system development | 488.984 | 612 | 1.06E-11 | up |
| signal transduction | 925.002 | 1078 | 3.14E-10 | up |
| adenyl ribonucleotide binding | 613.137 | 740 | 4.54E-10 | up |
| cytoskeletal protein binding | 208.475 | 285 | 4.54E-10 | up |
| ATP binding | 603.391 | 729 | 4.77E-10 | up |
| cellular developmental process | 798.731 | 939 | 8.78E-10 | up |
| neurogenesis | 269.492 | 354 | 1.28E-09 | up |
| cell communication | 696.188 | 827 | 1.30E-09 | up |
| cognition | 355.933 | 262 | 1.43E-09 | down |
| phosphotransferase activity, alcohol group as acceptor | 294.916 | 382 | 2.03E-09 | up |
| neuron differentiation | 227.967 | 305 | 2.03E-09 | up |
| regulation of cellular biosynthetic process | 1227.55 | 1391 | 2.26E-09 | up |
| cellular component organization | 1149.16 | 1308 | 2.47E-09 | up |
| regulation of cellular metabolic process | 1521.19 | 1697 | 3.02E-09 | up |
| cell differentiation | 761.019 | 893 | 4.13E-09 | up |
| regulation of primary metabolic process | 1446.19 | 1615 | 7.66E-09 | up |
| regulation of biosynthetic process | 1236.44 | 1395 | 7.97E-09 | up |
| regulation of metabolic process | 1596.61 | 1771 | 7.97E-09 | up |
| adenyl nucleotide binding | 645.341 | 766 | 7.97E-09 | up |
| organ development | 804.239 | 937 | 7.97E-09 | up |
| regulation of macromolecule biosynthetic process | 1165.26 | 1320 | 8.08E-09 | up |
| generation of neurons | 250.848 | 328 | 1.06E-08 | up |
| protein amino acid phosphorylation | 335.17 | 423 | 1.44E-08 | up |
| anatomical structure morphogenesis | 580.51 | 693 | 1.81E-08 | up |
| purine nucleoside binding | 660.171 | 779 | 1.98E-08 | up |
| regulation of nucleobase, nucleoside, nucleotide and nucleic acid metabolic process | 1223.31 | 1377 | 2.14E-08 | up |
| regulation of nitrogen compound metabolic process | 1233.9 | 1388 | 2.16E-08 | up |
| regulation of gene expression | 1188.99 | 1340 | 2.60E-08 | up |
| regulation of macromolecule metabolic process | 1377.12 | 1537 | 2.60E-08 | up |
| nucleoside binding | 664.408 | 782 | 3.00E-08 | up |
| kinase activity | 319.069 | 403 | 3.00E-08 | up |
| regulation of transcription | 1061.02 | 1204 | 3.51E-08 | up |
| negative regulation of cellular process | 783.476 | 909 | 3.80E-08 | up |
| transcription | 1100.43 | 1245 | 3.91E-08 | up |
| enzyme linked receptor protein signaling pathway | 197.882 | 264 | 4.27E-08 | up |
| intracellular | 4577.55 | 4766 | 4.42E-08 | up |
| negative regulation of biological process | 855.511 | 985 | 4.59E-08 | up |
| transferase activity, transferring phosphorus-containing groups | 368.645 | 457 | 5.05E-08 | up |
| protein kinase activity | 247.458 | 320 | 6.96E-08 | up |
| regulation of signaling pathway | 419.069 | 512 | 6.96E-08 | up |
| regulation of cell communication | 481.357 | 580 | 8.05E-08 | up |
| protein tyrosine kinase activity | 58.4747 | 94 | 9.69E-08 | up |
| cell development | 336.018 | 419 | 9.69E-08 | up |
| cell surface receptor linked signaling pathway | 622.883 | 733 | 9.89E-08 | up |
| nucleoside-triphosphatase regulator activity | 175 | 235 | 1.53E-07 | up |
| cell projection | 318.221 | 398 | 1.54E-07 | up |
| primary metabolic process | 3091.53 | 3280 | 1.66E-07 | up |

| | | | | |
|---|---|---|---|---|
| cellular macromolecule metabolic process | 2304.24 | 2482 | 1.83E-07 | up |
| ribonucleotide binding | 750.849 | 868 | 1.90E-07 | up |
| purine ribonucleotide binding | 750.849 | 868 | 1.90E-07 | up |
| cellular component morphogenesis | 197.882 | 261 | 1.90E-07 | up |
| GTPase regulator activity | 171.187 | 230 | 1.93E-07 | up |
| macromolecule metabolic process | 2537.72 | 2718 | 2.32E-07 | up |
| cell junction | 223.729 | 290 | 2.62E-07 | up |
| neuron development | 166.526 | 223 | 4.82E-07 | up |
| plasma membrane part | 834.324 | 953 | 5.49E-07 | up |
| intracellular signaling pathway | 630.51 | 735 | 6.33E-07 | up |
| protein modification process | 729.663 | 841 | 6.47E-07 | up |
| protein domain specific binding | 161.865 | 217 | 6.47E-07 | up |
| small GTPase regulator activity | 116.949 | 164 | 6.57E-07 | up |
| actin cytoskeleton | 115.678 | 162 | 9.14E-07 | up |
| transmembrane receptor protein tyrosine kinase signaling pathway | 123.305 | 171 | 9.48E-07 | up |
| cellular biosynthetic process | 1751.7 | 1907 | 9.48E-07 | up |
| actin binding | 132.627 | 182 | 9.63E-07 | up |
| macromolecule modification | 763.137 | 875 | 9.68E-07 | up |
| phosphorylation | 466.527 | 556 | 1.04E-06 | up |
| intermediate filament cytoskeleton | 74.5765 | 39 | 1.07E-06 | down |
| cell morphogenesis | 180.085 | 237 | 1.11E-06 | up |
| synapse | 155.085 | 208 | 1.12E-06 | up |
| biosynthetic process | 1795.34 | 1949 | 1.59E-06 | up |
| cell projection organization | 192.373 | 250 | 1.97E-06 | up |
| transcription regulator activity | 619.493 | 719 | 1.97E-06 | up |
| neuron projection | 154.238 | 206 | 1.98E-06 | up |
| nucleotide binding | 914.409 | 1032 | 2.06E-06 | up |
| purine nucleotide binding | 783.9 | 894 | 2.06E-06 | up |
| intermediate filament | 70.7629 | 37 | 2.26E-06 | down |
| cellular macromolecule biosynthetic process | 1421.61 | 1561 | 2.38E-06 | up |
| negative regulation of nucleobase, nucleoside, nucleotide and nucleic acid metabolic process | 236.865 | 300 | 2.38E-06 | up |
| macromolecule biosynthetic process | 1450 | 1590 | 2.57E-06 | up |
| transmembrane receptor activity | 493.645 | 407 | 3.45E-06 | down |
| negative regulation of nitrogen compound metabolic process | 239.407 | 302 | 3.45E-06 | up |
| cytoskeleton organization | 214.831 | 274 | 3.91E-06 | up |
| transmembrane receptor protein kinase activity | 34.7458 | 59 | 3.91E-06 | up |
| actin cytoskeleton organization | 115.255 | 159 | 3.99E-06 | up |
| intracellular part | 4440.69 | 4604 | 4.47E-06 | up |
| negative regulation of gene expression | 236.441 | 298 | 4.50E-06 | up |
| phosphorus metabolic process | 533.476 | 623 | 5.17E-06 | up |
| phosphate metabolic process | 533.476 | 623 | 5.17E-06 | up |
| negative regulation of transcription | 211.017 | 269 | 5.17E-06 | up |
| negative regulation of macromolecule biosynthetic process | 252.119 | 315 | 5.62E-06 | up |
| negative regulation of cellular biosynthetic process | 260.17 | 324 | 5.62E-06 | up |
| negative regulation of biosynthetic process | 263.984 | 328 | 6.23E-06 | up |
| actin filament-based process | 122.034 | 166 | 7.07E-06 | up |
| intracellular signal transduction | 500.425 | 586 | 7.75E-06 | up |
| central nervous system development | 194.068 | 248 | 1.19E-05 | up |
| cellular metabolic process | 3009.75 | 3170 | 1.36E-05 | up |
| positive regulation of cellular process | 844.494 | 950 | 1.40E-05 | up |
| neuron projection development | 135.17 | 180 | 1.54E-05 | up |
| transmembrane receptor protein tyrosine kinase activity | 27.5424 | 48 | 1.62E-05 | up |
| transcription repressor activity | 140.678 | 186 | 1.87E-05 | up |
| positive regulation of biological process | 927.545 | 1036 | 1.87E-05 | up |
| neuron projection morphogenesis | 103.39 | 142 | 2.47E-05 | up |
| receptor activity | 659.324 | 568 | 2.61E-05 | down |
| cytoskeleton | 576.273 | 663 | 2.71E-05 | up |
| cell morphogenesis involved in differentiation | 127.543 | 170 | 2.87E-05 | up |
| molecular_function | 6187.3 | 6290 | 3.15E-05 | up |
| localization | 1377.55 | 1501 | 3.65E-05 | up |
| cation binding | 1531.78 | 1660 | 3.72E-05 | up |
| ion binding | 1536.02 | 1664 | 3.95E-05 | up |
| transferase activity | 694.493 | 787 | 4.10E-05 | up |
| post-translational protein modification | 622.883 | 711 | 4.10E-05 | up |
| enzyme binding | 264.831 | 324 | 4.31E-05 | up |
| guanyl-nucleotide exchange factor activity | 62.712 | 92 | 5.07E-05 | up |
| keratin filament | 34.7458 | 14 | 5.12E-05 | down |
| transmission of nerve impulse | 175.848 | 224 | 5.41E-05 | up |

| | | | | |
|---|---|---|---|---|
| metal ion binding | 1514.83 | 1640 | 5.66E-05 | up |
| cytoplasm | 3124.16 | 3275 | 5.83E-05 | up |
| Ras protein signal transduction | 96.1867 | 132 | 5.85E-05 | up |
| metabolic process | 3390.69 | 3542 | 6.09E-05 | up |
| regulation of developmental process | 335.594 | 400 | 7.67E-05 | up |
| negative regulation of metabolic process | 368.221 | 435 | 8.98E-05 | up |
| regulation of signaling process | 324.153 | 387 | 9.09E-05 | up |
| negative regulation of cellular metabolic process | 335.17 | 399 | 9.09E-05 | up |
| structural constituent of ribosome | 63.5595 | 36 | 9.56E-05 | down |
| cell part morphogenesis | 123.729 | 163 | 0.000110957 | up |
| synaptic transmission | 150.848 | 194 | 0.000113058 | up |
| regulation of signal transduction | 322.035 | 384 | 0.000113058 | up |
| synapse part | 114.407 | 152 | 0.000123464 | up |
| zinc ion binding | 792.375 | 885 | 0.000130463 | up |
| cell morphogenesis involved in neuron differentiation | 100.848 | 136 | 0.000138837 | up |
| nucleobase, nucleoside, nucleotide and nucleic acid metabolic process | 1731.78 | 1857 | 0.000143923 | up |
| negative regulation of transcription from RNA polymerase II promoter | 121.61 | 160 | 0.000145064 | up |
| negative regulation of macromolecule metabolic process | 342.374 | 405 | 0.000160444 | up |
| chemokine activity | 19.4916 | 5 | 0.000163063 | down |
| regulation of small GTPase mediated signal transduction | 109.746 | 146 | 0.000167297 | up |
| small GTPase mediated signal transduction | 179.238 | 225 | 0.000179988 | up |
| regulation of transcription from RNA polymerase II promoter | 315.255 | 375 | 0.000186567 | up |
| axonogenesis | 91.1019 | 124 | 0.000188456 | up |
| regulation of multicellular organismal process | 449.577 | 520 | 0.000188456 | up |
| negative regulation of RNA metabolic process | 173.306 | 218 | 0.000205189 | up |
| regulation of cellular component organization | 226.272 | 277 | 0.000208185 | up |
| negative regulation of transcription, DNA-dependent | 170.763 | 215 | 0.000216551 | up |
| nitrogen compound metabolic process | 1897.04 | 2023 | 0.000218935 | up |
| cell projection morphogenesis | 118.221 | 155 | 0.000248674 | up |
| transcription from RNA polymerase II promoter | 381.781 | 446 | 0.000254306 | up |
| cellular nitrogen compound metabolic process | 1850.85 | 1974 | 0.000290307 | up |
| ribosome | 78.8137 | 50 | 0.000343906 | down |
| growth factor binding | 46.1866 | 69 | 0.000365847 | up |
| cytokine activity | 84.746 | 55 | 0.00037333 | down |
| transition metal ion binding | 903.392 | 995 | 0.000449456 | up |
| chemokine receptor binding | 21.6102 | 7 | 0.000451106 | down |
| intracellular organelle | 3780.52 | 3914 | 0.000640765 | up |
| Rho protein signal transduction | 51.695 | 75 | 0.000682657 | up |
| organelle | 3786.45 | 3918 | 0.00081933 | up |
| regulation of biological quality | 706.358 | 786 | 0.00081933 | up |
| protein serine/threonine kinase activity | 176.272 | 218 | 0.000826752 | up |
| nucleus | 2092.8 | 2213 | 0.000869223 | up |
| nucleic acid metabolic process | 1498.31 | 1606 | 0.000871293 | up |
| cell leading edge | 69.4917 | 96 | 0.000872435 | up |
| organ morphogenesis | 275.848 | 327 | 0.000962073 | up |
| neurological system process | 499.577 | 433 | 0.00100587 | down |
| DNA binding | 928.816 | 1017 | 0.00104107 | up |
| cellular component movement | 271.611 | 322 | 0.00108263 | up |
| Rho guanyl-nucleotide exchange factor activity | 30.5085 | 48 | 0.00109624 | up |
| regulation of cell proliferation | 353.391 | 410 | 0.00121709 | up |
| protein metabolic process | 1194.49 | 1291 | 0.00126102 | up |
| brain development | 132.627 | 168 | 0.00128121 | up |
| lamellipodium | 33.0509 | 51 | 0.00135841 | up |
| SH3 domain binding | 40.2543 | 60 | 0.00137172 | up |
| cell proliferation | 482.205 | 547 | 0.00137549 | up |
| postsynaptic membrane | 65.2544 | 90 | 0.00159079 | up |
| defense response | 289.831 | 240 | 0.00166507 | down |
| regulation of Ras protein signal transduction | 94.4918 | 124 | 0.0016668 | up |
| keratinization | 16.9492 | 5 | 0.00175937 | down |
| microspike assembly | 10.1695 | 20 | 0.00180725 | up |
| positive regulation of gene expression | 262.289 | 310 | 0.00191211 | up |
| generation of a signal involved in cell-cell signaling | 77.5426 | 104 | 0.00202369 | up |
| signal release | 77.5426 | 104 | 0.00202369 | up |
| positive regulation of transcription | 248.729 | 295 | 0.002057 | up |
| ribonucleoprotein complex | 200.848 | 160 | 0.00227761 | down |
| Ras guanyl-nucleotide exchange factor activity | 36.017 | 54 | 0.00248177 | up |
| regulation of localization | 307.628 | 358 | 0.00257389 | up |
| establishment of localization | 1190.26 | 1282 | 0.00257389 | up |

| | | | | |
|---|---|---|---|---|
| transcription factor activity | 389.831 | 446 | 0.00263505 | up |
| cytosol | 542.798 | 608 | 0.00272645 | up |
| Golgi apparatus part | 222.034 | 265 | 0.00272645 | up |
| cell part | 6024.17 | 6110 | 0.00278785 | up |
| cell | 6024.59 | 6110 | 0.00298509 | up |
| programmed cell death | 468.645 | 529 | 0.00312705 | up |
| postsynaptic density | 33.0509 | 50 | 0.00312705 | up |
| filopodium assembly | 9.74579 | 19 | 0.0033121 | up |
| apoptosis | 465.255 | 525 | 0.00343068 | up |
| transcription factor binding | 218.221 | 260 | 0.00355184 | up |
| sensory organ development | 104.661 | 134 | 0.00357628 | up |
| positive regulation of cellular biosynthetic process | 309.747 | 359 | 0.00358443 | up |
| skeletal muscle fiber development | 19.9153 | 33 | 0.00363843 | up |
| vasculature development | 144.916 | 179 | 0.00390102 | up |
| regulation of transcription, DNA-dependent | 725.849 | 798 | 0.00393044 | up |
| cellular component assembly | 420.764 | 477 | 0.00409084 | up |
| gene expression | 1531.78 | 1629 | 0.00412971 | up |
| polysaccharide biosynthetic process | 25.4238 | 40 | 0.00422276 | up |
| transport | 1175.43 | 1263 | 0.0043532 | up |
| transcription activator activity | 179.661 | 217 | 0.00450886 | up |
| intracellular membrane-bounded organelle | 3388.57 | 3505 | 0.0046548 | up |
| cell death | 513.137 | 574 | 0.00472664 | up |
| response to stimulus | 1492.8 | 1398 | 0.00477827 | down |
| digestive system development | 27.1187 | 42 | 0.00481737 | up |
| regulation of cellular component biogenesis | 63.5595 | 86 | 0.00496514 | up |
| calmodulin binding | 59.3222 | 81 | 0.00498322 | up |
| death | 514.408 | 575 | 0.00498322 | up |
| muscle cell differentiation | 60.1696 | 82 | 0.00498322 | up |
| positive regulation of nitrogen compound metabolic process | 291.526 | 338 | 0.00507745 | up |
| cellular protein metabolic process | 1005.51 | 1086 | 0.00558053 | up |
| transcription, DNA-dependent | 780.087 | 852 | 0.00596393 | up |
| blood vessel development | 141.526 | 174 | 0.00604662 | up |
| non-membrane spanning protein tyrosine kinase activity | 16.5255 | 28 | 0.00604662 | up |
| enzyme regulator activity | 353.814 | 404 | 0.00604662 | up |
| membrane-bounded organelle | 3391.53 | 3505 | 0.00626172 | up |
| Golgi membrane | 184.322 | 221 | 0.00634066 | up |
| ribosomal subunit | 48.7289 | 30 | 0.00634066 | down |
| regulation of Rho protein signal transduction | 45.3391 | 64 | 0.00634066 | up |
| regulation of peptide secretion | 25.8475 | 40 | 0.00649475 | up |
| cytokine receptor binding | 77.5426 | 54 | 0.00649475 | down |
| endocytosis | 113.983 | 143 | 0.00649475 | up |
| membrane invagination | 113.983 | 143 | 0.00649475 | up |
| RNA biosynthetic process | 781.782 | 853 | 0.00649475 | up |
| regulation of RNA metabolic process | 746.188 | 816 | 0.00649475 | up |
| regulation of peptide transport | 25.8475 | 40 | 0.00649475 | up |
| Golgi apparatus | 365.679 | 416 | 0.0068056 | up |
| regulation of cell differentiation | 234.323 | 275 | 0.00706815 | up |
| enzyme activator activity | 141.102 | 173 | 0.00710553 | up |
| camera-type eye morphogenesis | 22.034 | 35 | 0.00731679 | up |
| cell-cell signaling | 299.577 | 345 | 0.00754083 | up |
| positive regulation of transcription, DNA-dependent | 213.56 | 252 | 0.00818562 | up |
| cation channel activity | 111.865 | 140 | 0.00838208 | up |
| adherens junction | 61.0171 | 82 | 0.00843455 | up |
| heart development | 102.119 | 129 | 0.00849981 | up |
| GTPase activator activity | 91.5256 | 117 | 0.00857054 | up |
| nuclear membrane | 53.39 | 73 | 0.00868155 | up |
| mitochondrial inner membrane | 118.221 | 90 | 0.00875483 | down |
| positive regulation of macromolecule biosynthetic process | 291.95 | 336 | 0.00927427 | up |
| positive regulation of biosynthetic process | 314.408 | 360 | 0.00929038 | up |
| muscle structure development | 128.39 | 158 | 0.00986505 | up |
| identical protein binding | 289.407 | 333 | 0.00992527 | up |
| regulation of cell morphogenesis | 66.5256 | 88 | 0.0103348 | up |
| catalytic activity | 2111.87 | 2211 | 0.0105412 | up |
| peptide secretion | 37.712 | 54 | 0.0105444 | up |
| positive regulation of cellular metabolic process | 414.832 | 466 | 0.0107462 | up |
| mitochondrial part | 246.611 | 207 | 0.0107462 | down |
| MAPKKK cascade | 124.153 | 153 | 0.010876 | up |
| motor activity | 58.8984 | 79 | 0.0110637 | up |

| | | | | |
|---|---|---|---|---|
| cell projection part | 149.577 | 181 | 0.0113278 | up |
| renal system development | 46.1866 | 64 | 0.011458 | up |
| response to hormone stimulus | 188.984 | 224 | 0.0117538 | up |
| cytoplasmic membrane-bounded vesicle | 268.645 | 310 | 0.0118169 | up |
| muscle fiber development | 23.3051 | 36 | 0.0125631 | up |
| positive regulation of metabolic process | 436.442 | 488 | 0.0125976 | up |
| regulation of cellular component movement | 102.119 | 128 | 0.0125976 | up |
| cell maturation | 32.2035 | 47 | 0.0128999 | up |
| positive regulation of RNA metabolic process | 216.102 | 253 | 0.0132114 | up |
| multicellular organismal process | 1883.48 | 1977 | 0.0133071 | up |
| membrane raft | 63.5595 | 84 | 0.0135492 | up |
| positive regulation of protein kinase activity | 98.7291 | 124 | 0.0135492 | up |
| electron carrier activity | 63.9832 | 44 | 0.0137095 | down |
| regulation of peptide hormone secretion | 25.0001 | 38 | 0.0137863 | up |
| peptide hormone secretion | 36.4408 | 52 | 0.0140999 | up |
| signal complex assembly | 6.35595 | 13 | 0.0143864 | up |
| myosin complex | 25.8475 | 39 | 0.0143932 | up |
| cell-cell adherens junction | 15.678 | 26 | 0.0144144 | up |
| axon | 74.1527 | 96 | 0.0144144 | up |
| retina development in camera-type eye | 15.678 | 26 | 0.0144144 | up |
| ion channel activity | 157.627 | 189 | 0.0145071 | up |
| positive regulation of macromolecule metabolic process | 405.086 | 454 | 0.0146441 | up |
| kinesin binding | 5.08476 | 11 | 0.0146441 | up |
| developmental maturation | 42.373 | 59 | 0.0146441 | up |
| positive regulation of kinase activity | 102.543 | 128 | 0.0146441 | up |
| sequence-specific DNA binding | 263.984 | 304 | 0.0146441 | up |
| nucleus localization | 5.08476 | 11 | 0.0146441 | up |
| kidney development | 44.9154 | 62 | 0.0146639 | up |
| actin filament bundle | 12.7119 | 22 | 0.0146639 | up |
| negative regulation of cell proliferation | 160.594 | 192 | 0.0154042 | up |
| large ribosomal subunit | 25.4238 | 13 | 0.0155624 | down |
| neurotransmitter secretion | 21.1865 | 33 | 0.0156289 | up |
| cytoplasmic vesicle | 283.899 | 325 | 0.0156289 | up |
| positive regulation of transcription from RNA polymerase II promoter | 165.255 | 197 | 0.0157232 | up |
| cell migration | 191.95 | 226 | 0.0158794 | up |
| positive regulation of nucleobase, nucleoside, nucleotide and nucleic acid metabolic process | 283.052 | 324 | 0.0158794 | up |
| gated channel activity | 127.966 | 156 | 0.0158819 | up |
| voltage-gated ion channel activity | 78.8137 | 101 | 0.0159041 | up |
| voltage-gated channel activity | 78.8137 | 101 | 0.0159041 | up |
| mitochondrial membrane part | 49.1527 | 32 | 0.0163091 | down |
| cell motility | 205.933 | 241 | 0.0163091 | up |
| localization of cell | 205.933 | 241 | 0.0163091 | up |
| dendrite | 77.1188 | 99 | 0.0163145 | up |
| membrane-bounded vesicle | 276.696 | 317 | 0.0163611 | up |
| regulation of phosphorylation | 219.916 | 256 | 0.0166445 | up |
| regulation of insulin secretion | 22.8814 | 35 | 0.0171696 | up |
| cation channel complex | 57.2035 | 76 | 0.0176259 | up |
| positive regulation of signaling pathway | 160.17 | 191 | 0.0176485 | up |
| muscle organ development | 107.627 | 133 | 0.018396 | up |
| nucleosome | 22.4577 | 11 | 0.0183982 | down |
| integral to Golgi membrane | 18.2204 | 29 | 0.0183982 | up |
| axon guidance | 40.2543 | 56 | 0.0189296 | up |
| mitochondrion | 516.103 | 464 | 0.0209029 | down |
| basolateral plasma membrane | 94.4918 | 118 | 0.0209029 | up |
| regulation of cellular component size | 142.373 | 171 | 0.0209029 | up |
| regulation of locomotion | 102.543 | 127 | 0.0209029 | up |
| nucleosome assembly | 28.8136 | 16 | 0.0209803 | down |
| gland development | 84.746 | 107 | 0.0213283 | up |
| anatomical structure formation involved in morphogenesis | 194.916 | 228 | 0.0219369 | up |
| actin filament organization | 57.6273 | 76 | 0.0225958 | up |
| exocrine system development | 16.1017 | 26 | 0.023794 | up |
| presynaptic membrane | 16.1017 | 26 | 0.023794 | up |
| muscle cell development | 33.0509 | 47 | 0.0243686 | up |
| regulation of phosphate metabolic process | 228.814 | 264 | 0.0251133 | up |
| regulation of phosphorus metabolic process | 228.814 | 264 | 0.0251133 | up |
| receptor signaling protein tyrosine kinase activity | 5.93222 | 12 | 0.0251931 | up |
| cytoskeletal adaptor activity | 5.93222 | 12 | 0.0251931 | up |
| peptide transport | 41.5255 | 57 | 0.0253333 | up |

| | | | | |
|---|---|---|---|---|
| vesicle-mediated transport | 277.543 | 316 | 0.0253333 | up |
| synaptosome | 38.9831 | 54 | 0.0253333 | up |
| cell projection assembly | 41.5255 | 57 | 0.0253333 | up |
| response to oxygen levels | 64.8307 | 84 | 0.0253333 | up |
| epidermal cell differentiation | 34.7458 | 21 | 0.025725 | down |
| digestive tract development | 23.3051 | 35 | 0.025725 | up |
| protein-DNA complex assembly | 34.7458 | 21 | 0.025725 | down |
| negative regulation of cellular component organization | 74.5765 | 95 | 0.0261013 | up |
| regulation of cell migration | 92.3731 | 115 | 0.0261597 | up |
| keratinocyte differentiation | 30.9323 | 18 | 0.0264359 | down |
| vesicle | 296.611 | 336 | 0.0266344 | up |
| adenylate cyclase activity | 4.66103 | 10 | 0.0268905 | up |
| platelet-derived growth factor binding | 4.66103 | 10 | 0.0268905 | up |
| positive regulation of glycogen metabolic process | 4.66103 | 10 | 0.0268905 | up |
| negative regulation of protein complex disassembly | 17.7967 | 28 | 0.0270883 | up |
| cellular membrane organization | 183.051 | 214 | 0.0297167 | up |
| BMP signaling pathway | 27.5424 | 40 | 0.0298263 | up |
| substrate-specific channel activity | 161.865 | 191 | 0.0300338 | up |
| transcription cofactor activity | 150.848 | 179 | 0.0300451 | up |
| regulation of protein kinase activity | 156.356 | 185 | 0.0300451 | up |
| intrinsic to Golgi membrane | 19.4916 | 30 | 0.0307745 | up |
| SMAD binding | 19.4916 | 30 | 0.0307745 | up |
| oxidoreductase activity, acting on paired donors, with incorporation or reduction of molecular oxygen, NADH or NADPH as one donor, and incorporation of one atom of oxygen | 11.8644 | 4 | 0.0308427 | down |
| striated muscle cell development | 30.0848 | 43 | 0.0311685 | up |
| positive regulation of transferase activity | 106.356 | 130 | 0.0317232 | up |
| growth | 223.306 | 257 | 0.0317522 | up |
| protein depolymerization | 20.339 | 31 | 0.0322542 | up |
| urogenital system development | 61.8646 | 80 | 0.0323318 | up |
| G-protein signaling, coupled to cAMP nucleotide second messenger | 36.017 | 50 | 0.0323318 | up |
| carbohydrate transport | 38.5594 | 53 | 0.0323318 | up |
| regulation of hormone levels | 97.4579 | 120 | 0.0323318 | up |
| channel activity | 168.644 | 198 | 0.0323318 | up |
| gland morphogenesis | 36.017 | 50 | 0.0323318 | up |
| passive transmembrane transporter activity | 168.644 | 198 | 0.0323318 | up |
| chromatin assembly | 30.5085 | 18 | 0.0323318 | down |
| melanosome | 38.5594 | 53 | 0.0323318 | up |
| pigment granule | 38.5594 | 53 | 0.0323318 | up |
| canonical Wnt receptor signaling pathway | 33.4747 | 47 | 0.0323318 | up |
| membrane organization | 183.475 | 214 | 0.0323318 | up |
| response to other organism | 152.543 | 125 | 0.0325138 | down |
| respiratory chain | 26.695 | 15 | 0.0325138 | down |
| extracellular structure organization | 71.6103 | 91 | 0.0327608 | up |
| regulation of kinase activity | 162.289 | 191 | 0.0327608 | up |
| neuron recognition | 8.89833 | 16 | 0.0343323 | up |
| regulation of cytoskeleton organization | 57.6273 | 75 | 0.0343323 | up |
| positive regulation of cell differentiation | 108.475 | 132 | 0.0344912 | up |
| stress fiber | 11.8644 | 20 | 0.034567 | up |
| myelin sheath | 6.77968 | 13 | 0.034567 | up |
| cellular component maintenance | 11.8644 | 20 | 0.034567 | up |
| anchoring junction | 68.2205 | 87 | 0.034955 | up |
| cell adhesion | 333.052 | 373 | 0.0353505 | up |
| cellular localization | 497.035 | 545 | 0.0359784 | up |
| mitochondrial envelope | 175 | 146 | 0.036354 | down |
| regulation of anatomical structure morphogenesis | 130.509 | 156 | 0.036584 | up |
| regulation of neuron differentiation | 75.4239 | 95 | 0.036767 | up |
| response to endogenous stimulus | 211.017 | 243 | 0.0372812 | up |
| biological adhesion | 333.475 | 373 | 0.0386452 | up |
| blood vessel morphogenesis | 122.458 | 147 | 0.0388013 | up |
| positive regulation of peptide secretion | 12.7119 | 21 | 0.0388679 | up |
| nucleosome organization | 32.6272 | 20 | 0.0389177 | down |
| regulation of molecular function | 444.069 | 489 | 0.0399146 | up |
| regulation of anatomical structure size | 169.492 | 198 | 0.0404847 | up |
| protein homooligomerization | 44.0679 | 59 | 0.0405828 | up |
| positive regulation of cell communication | 174.153 | 203 | 0.0406981 | up |
| glycosaminoglycan biosynthetic process | 9.74579 | 17 | 0.040824 | up |
| voltage-gated cation channel activity | 59.7459 | 77 | 0.0408563 | up |
| ear development | 42.373 | 57 | 0.0408697 | up |

| | | | | |
|---|---|---|---|---|
| ruffle | 31.356 | 44 | 0.0412167 | up |
| insulin secretion | 31.356 | 44 | 0.0412167 | up |
| focal adhesion | 36.4408 | 50 | 0.0416032 | up |
| cell junction organization | 33.8984 | 47 | 0.0416032 | up |
| tube development | 141.949 | 168 | 0.0416032 | up |
| eye morphogenesis | 34.7458 | 48 | 0.0416032 | up |
| negative regulation of signaling pathway | 112.712 | 136 | 0.0418743 | up |
| organelle organization | 610.595 | 662 | 0.0420762 | up |
| filopodium | 13.5594 | 22 | 0.0420762 | up |
| neuronal cell body | 75.8476 | 95 | 0.04353 | up |
| cell body | 75.8476 | 95 | 0.04353 | up |
| semaphorin receptor activity | 2.96611 | 7 | 0.0441824 | up |
| retina morphogenesis in camera-type eye | 7.62714 | 14 | 0.0445754 | up |
| microtubule | 115.678 | 139 | 0.0454255 | up |
| actin filament binding | 20.7628 | 31 | 0.0455415 | up |
| nuclear envelope | 95.7629 | 117 | 0.0463311 | up |
| protein complex assembly | 241.95 | 275 | 0.0463311 | up |
| protein complex biogenesis | 241.95 | 275 | 0.0463311 | up |
| aminoglycan biosynthetic process | 10.5932 | 18 | 0.0465744 | up |
| cytochrome-c oxidase activity | 9.74579 | 3 | 0.0468106 | down |
| response to biotic stimulus | 188.984 | 160 | 0.0468106 | down |
| heme-copper terminal oxidase activity | 9.74579 | 3 | 0.0468106 | down |
| oxidoreductase activity, acting on heme group of donors | 9.74579 | 3 | 0.0468106 | down |
| oxidoreductase activity, acting on heme group of donors, oxygen as acceptor | 9.74579 | 3 | 0.0468106 | down |
| ion channel complex | 87.7121 | 108 | 0.0469103 | up |
| extracellular region | 799.578 | 743 | 0.0472259 | down |
| clathrin coat of coated pit | 4.2373 | 9 | 0.0472259 | up |
| positive regulation of glycogen biosynthetic process | 4.2373 | 9 | 0.0472259 | up |
| regulation of cell death | 373.73 | 414 | 0.0477704 | up |
| transcription corepressor activity | 61.0171 | 78 | 0.0478799 | up |
| embryonic development | 261.018 | 295 | 0.0481942 | up |
| GTPase binding | 45.3391 | 60 | 0.0496326 | up |
