# Supplementary material for: Characterizing 5-hydroxymethylcytosine in human prefrontal cortex at single base resolution
Source: BMC Genomics. 2015 Sep 3;16(1):672. doi: 10.1186/s12864-015-1875-8 (PMC4559220; doi:10.1186/s12864-015-1875-8)
Supplement: Additional file 7: — is a table containing the GO terms from a cluster analysis using 5hmC sites unique to females. (PDF 71 kb) [file 12864_2015_1875_MOESM7_ESM.pdf]

Supplementary Table S4 - Cluster analysis in 5hmC sites unique to females

GO Term	expected	observed	p-value (fdr)	enrichment
olfactory receptor activity	91.4081	23	6.00E-18	down
sensory perception of smell	95.9123	29	5.08E-16	down
sensory perception of chemical stimulus	106.245	37	2.82E-15	down
transcription regulator activity	387.359	526	4.87E-14	up
transcription factor activity	243.755	354	1.97E-13	up
regulation of nitrogen compound metabolic process	771.538	944	2.13E-12	up
regulation of nucleobase, nucleoside, nucleotide and nucleic acid metabolic process	764.914	936	2.44E-12	up
regulation of transcription	663.438	822	7.38E-12	up
sequence-specific DNA binding	165.065	251	8.74E-12	up
transcription	688.078	846	1.65E-11	up
regulation of gene expression	743.453	906	1.65E-11	up
regulation of primary metabolic process	904.278	1074	7.53E-11	up
regulation of cellular biosynthetic process	767.563	926	1.08E-10	up
regulation of macromolecule biosynthetic process	728.616	882	1.91E-10	up
regulation of RNA metabolic process	466.579	594	2.16E-10	up
regulation of biosynthetic process	773.127	929	2.34E-10	up
regulation of transcription, DNA-dependent	453.861	579	2.61E-10	up
transcription, DNA-dependent	487.775	616	3.36E-10	up
RNA biosynthetic process	488.835	617	3.41E-10	up
DNA binding	580.773	716	7.73E-10	up
biological regulation	1774.64	1967	8.70E-10	up
regulation of cellular metabolic process	951.174	1112	1.45E-09	up
regulation of cellular process	1589.97	1776	1.53E-09	up
regulation of metabolic process	998.336	1160	2.25E-09	up
organ morphogenesis	172.483	249	2.94E-09	up
regulation of macromolecule metabolic process	861.091	1013	3.26E-09	up
binding	3124.57	3294	4.38E-09	up
regulation of biological process	1672.9	1855	5.13E-09	up
negative regulation of gene expression	147.843	215	3.01E-08	up
negative regulation of transcription	131.946	195	4.36E-08	up
G-protein coupled receptor activity	200.833	130	5.02E-08	down
transcription from RNA polymerase II promoter	238.721	321	5.18E-08	up
anatomical structure morphogenesis	362.983	462	5.47E-08	up
nervous system development	305.754	397	6.59E-08	up
cellular macromolecule biosynthetic process	888.911	1029	1.05E-07	up
brain development	82.9297	132	1.28E-07	up
negative regulation of transcription, DNA-dependent	106.775	162	1.28E-07	up
nucleobase, nucleoside, nucleotide and nucleic acid metabolic process	1082.86	1231	1.50E-07	up
intermediate filament cytoskeleton	46.6314	15	1.88E-07	down
central nervous system development	121.348	179	2.10E-07	up
negative regulation of RNA metabolic process	108.365	163	2.14E-07	up
neurogenesis	168.509	235	2.71E-07	up
negative regulation of cellular process	489.895	597	2.71E-07	up
neuron differentiation	142.544	204	2.78E-07	up
intermediate filament	44.2468	14	3.07E-07	down
sensory perception	197.389	131	3.07E-07	down
macromolecule biosynthetic process	906.663	1041	4.44E-07	up
generation of neurons	156.851	220	4.53E-07	up
negative regulation of transcription from RNA polymerase II promoter	76.041	121	4.89E-07	up
nucleic acid binding	810.75	939	4.90E-07	up
regulation of transcription from RNA polymerase II promoter	197.124	267	4.90E-07	up
anatomical structure development	740.538	864	5.41E-07	up
DNA regulatory region binding	32.8539	63	5.72E-07	up
cellular process	2947.85	3100	5.95E-07	up
negative regulation of nitrogen compound metabolic process	149.697	210	8.78E-07	up
system development	668.472	785	8.92E-07	up
negative regulation of nucleobase, nucleoside, nucleotide and nucleic acid metabolic process	148.108	208	8.99E-07	up
cell fate commitment	37.0932	68	1.43E-06	up
negative regulation of biological process	534.936	639	1.69E-06	up
cellular nitrogen compound metabolic process	1157.31	1296	1.76E-06	up
promoter binding	31.7941	60	2.39E-06	up
organ development	502.877	603	2.39E-06	up
cell development	210.106	278	2.43E-06	up
nitrogen compound metabolic process	1186.19	1323	3.13E-06	up
embryonic morphogenesis	90.6133	136	3.38E-06	up
nucleic acid metabolic process	936.867	1063	3.38E-06	up
keratin filament	21.726	3	3.43E-06	down
cellular biosynthetic process	1095.31	1228	3.47E-06	up
RNA metabolic process	651.515	760	4.62E-06	up
developmental process	886.261	1008	4.93E-06	up
multicellular organismal development	807.836	925	5.45E-06	up
tissue morphogenesis	76.041	117	5.56E-06	up
embryonic organ development	56.4346	92	5.75E-06	up
protein binding	2090.46	2239	6.46E-06	up
biosynthetic process	1122.6	1253	6.47E-06	up
negative regulation of biosynthetic process	165.065	223	7.76E-06	up
negative regulation of cellular biosynthetic process	162.68	220	8.52E-06	up
positive regulation of gene expression	164.005	220	1.71E-05	up
cell differentiation	475.852	566	1.78E-05	up

cellular developmental process	499.433	591	1.99E-05	up
cognition	222.559	162	2.03E-05	down
negative regulation of macromolecule biosynthetic process	157.646	212	2.17E-05	up
pattern specification process	73.9214	112	2.30E-05	up
embryonic development ending in birth or egg hatching	99.3567	143	2.38E-05	up
cell morphogenesis involved in differentiation	79.7503	119	2.53E-05	up
embryonic development	163.21	218	2.53E-05	up
morphogenesis of an epithelium	58.2892	92	2.82E-05	up
gene expression	957.798	1074	2.82E-05	up
chordate embryonic development	98.2969	141	3.38E-05	up
transcription repressor activity	87.9638	128	4.50E-05	up
positive regulation of macromolecule biosynthetic process	182.551	238	6.16E-05	up
positive regulation of transcription, DNA-dependent	133.535	181	7.73E-05	up
positive regulation of transcription	155.526	206	9.42E-05	up
nucleus	1308.59	1430	9.68E-05	up
cell communication	435.315	515	0.000111017	up
molecular_function	3868.82	3954	0.00012019	up
central nervous system neuron differentiation	19.3414	38	0.000143767	up
regulation of cell proliferation	220.969	279	0.000144971	up
positive regulation of transcription from RNA polymerase II promoter	103.331	144	0.000153035	up
positive regulation of cellular biosynthetic process	193.679	248	0.000159592	up
positive regulation of RNA metabolic process	135.125	181	0.000165463	up
positive regulation of biosynthetic process	196.594	251	0.000174735	up
regionalization	58.2892	89	0.000191672	up
cell projection organization	120.288	163	0.000228592	up
cell morphogenesis	112.604	154	0.000228674	up
zinc ion binding	495.459	576	0.000254297	up
forebrain neuron differentiation	3.70932	12	0.000277761	up
cellular component morphogenesis	123.732	166	0.000350543	up
heart looping	8.74339	21	0.000357303	up
embryonic heart tube morphogenesis	8.74339	21	0.000357303	up
generation of neurons in the forebrain	4.76912	14	0.000357303	up
neuron development	104.126	143	0.000358854	up
epithelial tube morphogenesis	36.2983	60	0.000365267	up
cellular macromolecule metabolic process	1440.8	1556	0.000401003	up
tissue development	215.405	269	0.000475655	up
response to stimulus	933.423	836	0.000553792	down
regulation of cell development	70.477	102	0.000566494	up
heart morphogenesis	25.4353	45	0.000566532	up
regulation of gene-specific transcription from RNA polymerase II promoter	42.3922	67	0.000655886	up
gene-specific transcription from RNA polymerase II promoter	42.3922	67	0.000655886	up
epithelium development	96.9721	133	0.000729033	up
embryonic organ morphogenesis	36.2983	59	0.000744248	up
regulation of signaling pathway	262.037	319	0.000746684	up
gland development	52.9902	80	0.000775305	up
regulation of gene-specific transcription	58.0243	86	0.000874344	up
positive regulation of nitrogen compound metabolic process	182.286	230	0.000880128	up
substrate-specific transmembrane transporter activity	210.106	261	0.000883889	up
positive regulation of nucleobase, nucleoside, nucleotide and nucleic acid metabolic process	176.987	224	0.000883889	up
neuron projection morphogenesis	64.6481	94	0.000886734	up
RNA polymerase II transcription factor activity	61.4687	90	0.000953722	up
negative regulation of metabolic process	230.243	283	0.000954284	up
anatomical structure formation involved in morphogenesis	121.878	161	0.0010206	up
negative regulation of transforming growth factor beta receptor signaling pathway	7.41863	18	0.00109399	up
cytokine activity	52.9902	29	0.00110688	down
B cell mediated immunity	18.8115	5	0.00110688	down
signaling	923.62	1017	0.00122047	up
tube development	88.7586	122	0.00125285	up
signaling pathway	660.258	742	0.0013052	up
immunoglobulin mediated immune response	18.5466	5	0.0013625	down
forebrain development	45.8365	70	0.00141578	up
regulation of developmental process	209.841	259	0.00141578	up
adaptive immune response	30.7343	13	0.00143871	down
transcription activator activity	112.339	149	0.0014684	up
cell projection morphogenesis	73.9214	104	0.00150122	up
cell part morphogenesis	77.3657	108	0.0015653	up
apoptosis	290.916	347	0.00179872	up
transmembrane receptor activity	308.668	253	0.0018094	down
ribonuclease activity	16.427	4	0.00187804	down
neuron projection development	84.5194	116	0.00190456	up
programmed cell death	293.036	349	0.00190565	up
cell death	320.856	379	0.00196994	up
adaptive immune response based on somatic recombination of immune receptors built from immunoglobulin superfamily	30.2044	13	0.00198298	down
channel activity	105.451	140	0.00217046	up
passive transmembrane transporter activity	105.451	140	0.00217046	up
substrate-specific channel activity	101.211	135	0.00225876	up
neuron fate commitment	10.3331	22	0.00226973	up
death	321.651	379	0.00237913	up
cell proliferation	301.514	357	0.0025107	up
macromolecule metabolic process	1586.79	1690	0.00271504	up
regulation of cell differentiation	146.518	186	0.00282417	up

placenta development	23.3157	40	0.0028828	up
positive regulation of macromolecule metabolic process	253.293	304	0.00288681	up
negative regulation of transmembrane receptor protein serine/threonine kinase signaling pathway	11.9228	24	0.00344997	up
production of molecular mediator of immune response	17.2218	5	0.00376413	down
heart development	63.8532	90	0.00382269	up
cell morphogenesis involved in neuron differentiation	63.0584	89	0.00390764	up
transmembrane transporter activity	230.772	278	0.00420888	up
cell fate specification	11.3929	23	0.00433784	up
endocrine system development	21.461	37	0.00433784	up
positive regulation of cellular process	528.048	596	0.00433784	up
neurotransmitter:sodium symporter activity	5.03407	13	0.00443226	up
midbrain development	5.03407	13	0.00443226	up
respiratory system development	29.1446	47	0.00445755	up
ion channel activity	98.5618	130	0.00473314	up
negative regulation of macromolecule metabolic process	214.08	259	0.00497652	up
negative regulation of cellular metabolic process	209.576	254	0.00503681	up
positive regulation of metabolic process	272.9	323	0.00504009	up
lung development	26.2302	43	0.00521148	up
liver development	17.2218	31	0.00523005	up
lymphocyte mediated immunity	28.6147	13	0.00523005	down
cell surface receptor linked signaling pathway	389.478	448	0.00523005	up
specific RNA polymerase II transcription factor activity	10.863	22	0.00523012	up
enhancer binding	10.863	22	0.00523012	up
positive regulation of cellular metabolic process	259.387	308	0.00532866	up
dorsal spinal cord development	2.91446	9	0.00533192	up
sensory organ development	65.4429	91	0.00535817	up
positive regulation of gene-specific transcription	38.153	58	0.00535817	up
cellular metabolic process	1881.95	1982	0.00535817	up
positive regulation of cell development	23.3157	39	0.00576544	up
ion transmembrane transporter activity	183.081	224	0.00604011	up
embryonic heart tube development	11.6578	23	0.00604011	up
cellular component organization	718.547	793	0.00607759	up
hepaticobiliary system development	17.4868	31	0.00692181	up
endoplasmic reticulum membrane	159.236	123	0.00709925	down
regulation of multicellular organismal process	281.113	330	0.0074974	up
regulation of transmembrane receptor protein serine/threonine kinase signaling pathway	24.3755	40	0.00759509	up
mesenchymal cell differentiation	19.0765	33	0.00771795	up
regulation of anatomical structure morphogenesis	81.6049	109	0.00786336	up
tube morphogenesis	66.2378	91	0.00813069	up
endocrine pancreas development	5.29902	13	0.00824563	up
immunoglobulin production	12.7177	3	0.00879197	down
nuclear membrane-endoplasmic reticulum network	162.945	127	0.00879197	down
intracellular	2862.27	2954	0.00920504	up
positive regulation of cell proliferation	118.963	151	0.0097563	up
respiratory tube development	27.025	43	0.00993939	up
cation transmembrane transporter activity	139.629	174	0.0101976	up
leukocyte mediated immunity	35.5034	19	0.0102651	down
anterior/posterior pattern formation	40.0076	59	0.0109611	up
regulation of transforming growth factor beta receptor signaling pathway	13.5125	25	0.0109611	up
embryonic skeletal system development	21.726	36	0.0110321	up
mesenchyme development	21.726	36	0.0110321	up
receptor activity	412.264	358	0.0116855	down
neurotransmitter transporter activity	6.09388	14	0.0121835	up
regulation of cell communication	300.984	349	0.0122878	up
negative regulation of developmental process	76.5709	102	0.0123099	up
axonogenesis	56.9645	79	0.0128827	up
metal ion binding	947.2	1024	0.0130605	up
kinesin binding	3.17941	9	0.0138172	up
positive regulation of biological process	579.978	643	0.0140297	up
substrate-specific transporter activity	246.934	290	0.0147967	up
negative regulation of caspase activity	5.56397	13	0.0148403	up
positive regulation of gene-specific transcription from RNA polymerase II promoter	28.3498	44	0.0149832	up
mesenchymal cell development	18.2816	31	0.0155797	up
neurological system process	312.377	266	0.0155797	down
in utero embryonic development	59.0841	81	0.015891	up
endoderm development	6.88873	15	0.0161439	up
transition metal ion binding	564.876	626	0.0168005	up
embryonic placenta development	15.3672	27	0.0168913	up
epithelial cell development	10.3331	20	0.0174012	up
RNA polymerase II transcription factor activity, enhancer binding	10.3331	20	0.0174012	up
negative regulation of gene-specific transcription	23.8456	38	0.0178984	up
ion binding	960.448	1035	0.0179032	up
subsynaptic reticulum	185.466	150	0.0188305	down
plasma membrane part	521.689	580	0.0188681	up
negative regulation of gene-specific transcription from RNA polymerase II promoter	17.7517	30	0.0191895	up
transmembrane receptor protein serine/threonine kinase signaling pathway	43.452	62	0.01932	up
cation channel activity	69.9471	93	0.0204388	up
cation binding	957.798	1031	0.0209371	up
negative regulation of muscle cell differentiation	4.50417	11	0.0212889	up
regulation of mitochondrial membrane potential	4.50417	11	0.0212889	up
gland morphogenesis	22.5208	36	0.0216657	up

ear development	26.4951	41	0.0220263	up
formation of primary germ layer	12.7177	23	0.0232591	up
cytoskeleton organization	134.33	165	0.0244005	up
regulation of small GTPase mediated signal transduction	68.6223	91	0.0244005	up
endoribonuclease activity	11.3929	3	0.0245555	down
nucleosome organization	20.4012	9	0.0245555	down
positive regulation of immune response	41.3324	25	0.0248317	down
camera-type eye development	32.324	48	0.0248512	up
regulation of neurogenesis	57.4944	78	0.0249574	up
compartment pattern formation	1.32476	5	0.0250621	up
negative regulation of signaling pathway	70.477	93	0.025186	up
morphogenesis of a branching epithelium	29.1446	44	0.0258338	up
microtubule	72.3317	95	0.0263526	up
solute:cation symporter activity	22.7858	36	0.0263526	up
morphogenesis of a branching structure	34.1787	50	0.028466	up
mitochondrion	322.71	279	0.0297715	down
regulation of production of molecular mediator of immune response	11.1279	3	0.0298135	down
primary metabolic process	1933.08	2017	0.0298135	up
transcription factor complex	51.9304	71	0.030392	up
regulation of muscle cell differentiation	10.0681	19	0.030392	up
endoplasmic reticulum part	176.722	144	0.0303988	down
negative regulation of cell differentiation	62.2635	83	0.0305422	up
ion transport	212.491	249	0.0327876	up
membrane depolarization	10.863	20	0.033454	up
positive regulation of neuroblast proliferation	1.85466	6	0.0335201	up
enzyme linked receptor protein signaling pathway	123.732	152	0.0335201	up
negative regulation of response to extracellular stimulus	1.85466	6	0.0335201	up
negative regulation of response to nutrient levels	1.85466	6	0.0335201	up
intrinsic to plasma membrane	311.847	355	0.0344256	up
activation of immune response	29.1446	16	0.0344946	down
dendrite cytoplasm	2.38456	7	0.0344946	up
paraxial mesoderm morphogenesis	2.38456	7	0.0344946	up
endoplasmic reticulum	262.832	224	0.03466	down
spinal cord development	11.6578	21	0.0352067	up
defense response to virus	11.6578	21	0.0352067	up
macromolecular complex	821.878	758	0.0352122	down
activation of plasma proteins involved in acute inflammatory response	10.863	3	0.0353077	down
extracellular-glutamate-gated ion channel activity	4.76912	11	0.0358407	up
negative regulation of cell communication	88.2287	112	0.0367989	up
excitatory extracellular ligand-gated ion channel activity	12.4527	22	0.0372985	up
positive regulation of neurogenesis	18.5466	30	0.0373281	up
canonical Wnt receptor signaling pathway	20.9311	33	0.0383558	up
regulation of membrane potential	38.153	54	0.0405048	up
specific transcriptional repressor activity	10.3331	19	0.0409167	up
cardiac septum development	4.23922	10	0.0435341	up
epithelial cell morphogenesis	4.23922	10	0.0435341	up
chromatin assembly or disassembly	29.9395	17	0.0435341	down
excretion	15.6321	26	0.0435341	up
dorsal/ventral pattern formation	15.6321	26	0.0435341	up
pancreas development	11.1279	20	0.0435341	up
intracellular part	2776.69	2854	0.0435341	up
negative regulation of osteoblast differentiation	4.23922	10	0.0435341	up
negative regulation of cell proliferation	100.416	125	0.0444457	up
cell differentiation in spinal cord	6.88873	14	0.0448678	up
cellular response to extracellular stimulus	25.9652	39	0.0448678	up
lung morphogenesis	6.88873	14	0.0448678	up
gated channel activity	80.0152	102	0.0457961	up
microtubule-based process	71.2718	92	0.0474134	up
Wnt receptor signaling pathway	49.5459	67	0.0476228	up
regulation of nervous system development	62.5285	82	0.0476228	up
regulation of cellular component organization	141.484	170	0.0479425	up
nucleotide binding	571.765	625	0.0490977	up
regulation of cytoskeleton organization	36.0334	51	0.0490977	up
