# Supplementary material for: Characterizing 5-hydroxymethylcytosine in human prefrontal cortex at single base resolution
Source: BMC Genomics. 2015 Sep 3;16(1):672. doi: 10.1186/s12864-015-1875-8 (PMC4559220; doi:10.1186/s12864-015-1875-8)
Supplement: Additional file 8: — is a table containing the results of a differential analysis of gene expression in males and females using previously published RNA-Seq data [ 23 ]. (PDF 76 kb) [file 12864_2015_1875_MOESM8_ESM.pdf]

Supplementary Table S5 - Differential Gene Expression between males and females from Akula 2014

Gene	Ensembl ID	baseMean	log2FoldChange	p-value	Adjusted p-value
XIST	ENSG00000229807	27143.36991	2.461985605	7.95E-41	1.41E-37
ZFX	ENSG00000005889	2624.620477	0.653325343	6.60E-08	7.41E-05
ENPP2	ENSG00000136960	15757.83067	0.660906511	1.24E-05	0.011067117
OTOF	ENSG00000115155	328.8290401	0.652437267	5.26E-05	0.043171013
P2RY13	ENSG00000181631	379.5831507	0.728117741	7.21E-05	0.053046027
RAB3B	ENSG00000169213	6254.219996	0.467479666	0.00010749	0.076443359
GPR78	ENSG00000155269	37.47069932	0.688963595	0.000144745	0.099617065
ANP32A	ENSG00000140350	8694.593525	0.298110676	0.000173602	0.112236585
CASR	ENSG00000036828	15.02714533	0.593770968	0.000379131	0.23110759
RP11-887P2.1	NA	240.9953401	0.417055207	0.000455379	0.269875302
TSPEAR-AS1	NA	14.30414225	0.576199972	0.000620888	0.323088906
DPYSL5	ENSG00000157851	10506.37683	0.572657911	0.000649124	0.323726715
P2RY12	ENSG00000169313	1037.040065	0.610347192	0.000914919	0.368298098
ZNF311	NA	86.02362589	0.531919469	0.000949229	0.374938805
ENPP6	ENSG00000164303	1528.483458	0.551956906	0.001137008	0.4182424
RP5-1086L22.1	NA	7.626392227	0.547581362	0.001232039	0.441343141
TLR7	ENSG00000196664	153.0740006	0.593223329	0.00137614	0.458749028
SCUBE1	ENSG00000159307	154.5745393	0.570472418	0.001615427	0.486374754
SNRPF	ENSG00000139343	2813.099707	0.437070982	0.001641	0.486374754
TLR10	ENSG00000174123	133.2625248	0.58152872	0.001708958	0.486374754
PDGFRA	ENSG00000134853	3678.077454	0.514524031	0.001709778	0.486374754
KLK5	ENSG00000167754	279.2504958	0.521334798	0.002075901	0.558562712
RP5-1142A6.2	NA	51.9484078	0.502780507	0.002094447	0.558562712
CHP2	ENSG00000166869	37.78194704	0.56297248	0.002196848	0.57863901
KRT78	ENSG00000170423	15.42413528	0.520740054	0.00231392	0.594788978
C21orf90	NA	40.46686588	0.531837411	0.002446952	0.607330885
DDX3X	ENSG00000215301	22061.1329	0.309144083	0.002505044	0.607330885
PNPLA4	ENSG00000006757	1244.94564	0.342183814	0.00256818	0.607978826
AC007620.3	NA	581.0609945	0.369512939	0.002621704	0.607978826
KDM6A	ENSG00000147050	1955.385717	0.429192188	0.002675238	0.610155169
ATAD3C	ENSG00000215915	304.182738	0.527776476	0.002688286	0.610155169
JPX	ENSG00000225470	1840.28353	0.466073048	0.002876749	0.64605724
PRAP1	ENSG00000165828	32.38615068	0.543320268	0.003047306	0.662075851
SAMD4A	ENSG00000020577	3951.136295	0.27800941	0.00353087	0.724337689
CTD-2224J9.2	NA	505.1222983	0.482002857	0.003598383	0.73115706
SLC17A8	ENSG00000179520	178.3507783	0.433037873	0.003679348	0.740555507
STK32B	ENSG00000152953	330.924145	0.372103439	0.003835862	0.74796996
BACE2	ENSG00000182240	412.5463132	0.437281669	0.003857964	0.74796996
RP5-1166H10.2	NA	109.2080251	0.515898103	0.003884645	0.74796996
GSTA1	NA	51.33487349	0.45477709	0.003926536	0.74796996
C2orf27B	ENSG00000186825	1.882996047	0.326637124	0.004292509	NA
ABP1	NA	2.551134899	0.317358141	0.004345608	NA
CTC-497E21.4	NA	91.60312177	0.524812035	0.004485174	0.833870194
YPEL2	ENSG00000175155	7565.478143	0.329816679	0.004527984	0.833870194
CYP24A1	ENSG00000019186	130.7494167	0.471088755	0.004968264	0.872163305
LINC00323	ENSG00000226496	71.10712192	0.517004276	0.00506331	0.872163305

TNIP3	ENSG00000050730	129.1130568	0.503870629	0.005207316	0.872163305
HN1L	ENSG00000206053	1320.102785	0.500234746	0.005236107	0.872163305
CD244	ENSG00000122223	70.22415638	0.513257089	0.005488256	0.872163305
KLK8	ENSG00000129455	5.274137383	0.445704506	0.005638538	0.872163305
RPLP0P2	ENSG00000243742	324.0229044	0.461463885	0.005946932	0.901748041
COL4A5	ENSG00000188153	4463.436239	0.507970696	0.006145996	0.904978396
ZNF833P	ENSG00000197332	203.8243082	0.427033064	0.006150545	0.904978396
SHISA2	ENSG00000180730	118.8171545	0.497930487	0.006345424	0.919651571
MBOAT1	ENSG00000172197	157.0632312	0.498959763	0.006466571	0.919651571
EPCAM	ENSG00000119888	201.979206	0.504076732	0.006515873	0.919651571
EHD4	ENSG00000103966	698.3221135	0.31125339	0.006750332	0.928899261
EXT1	ENSG00000182197	2061.79168	0.266786429	0.006792045	0.928899261
SPHKAP	ENSG00000153820	4347.532055	0.414547441	0.007001526	0.946340411
GBP3	ENSG00000117226	222.9379592	0.4903279	0.007050603	0.946340411
COX7A2	ENSG00000112695	19312.3122	0.327014285	0.007148218	0.946512897
AC103965.1	NA	253.2491722	0.485268923	0.007187021	0.946512897
CYBB	NA	602.6553289	0.481054727	0.007471216	0.960779083
ADAM28	ENSG00000042980	555.4941457	0.471852217	0.00747372	0.960779083
RAI14	ENSG00000039560	913.8307518	0.453001328	0.007520302	0.960779083
NXPH4	ENSG00000182379	289.3536686	0.461783708	0.007552951	0.960779083
GPR34	ENSG00000171659	268.5332522	0.487805906	0.00765561	0.960779083
TMEM45A	ENSG00000181458	95.21055753	0.489416065	0.007783202	0.971079609
TBC1D3G	NA	14.80089695	0.307112073	0.008378694	0.999970281
MGC14436	NA	119.4624808	0.470781559	0.008476919	0.999970281
FABP6	ENSG00000170231	601.1241518	0.419060241	0.00902496	0.999970281
FIBCD1	ENSG00000130720	233.2909234	0.481772544	0.009167895	0.999970281
RMI2	ENSG00000175643	176.0445379	0.458604405	0.009875507	0.999970281
DIRAS2	ENSG00000165023	43549.48356	0.36525519	0.010072913	0.999970281
RP11-314A5.3	NA	38.82107501	0.473962458	0.010088355	0.999970281
MOBP	ENSG00000168314	54302.8112	0.475546045	0.010237905	0.999970281
CHCHD8	NA	1792.738692	0.253931365	0.010453216	0.999970281
ASPA	ENSG00000108381	5579.44652	0.467710844	0.010513637	0.999970281
RASGEF1B	ENSG00000138670	1270.568081	0.382962515	0.011501871	0.999970281
SSTR1	ENSG00000139874	3722.091489	0.421644865	0.011785945	0.999970281
TAC1	ENSG00000006128	1354.943521	0.433381756	0.012018211	0.999970281
AC108142.1	NA	72.35026106	0.384849049	0.01202885	0.999970281
LEMD1	ENSG00000186007	12.99215195	0.45974813	0.012188842	0.999970281
FCGR3A	ENSG00000203747	388.0338332	0.462471022	0.012323914	0.999970281
MYO16	ENSG00000041515	1755.648947	0.324442072	0.01239075	0.999970281
FOXP2	ENSG00000128573	1964.626379	0.319798882	0.012439119	0.999970281
XKR4	ENSG00000206579	2159.209803	0.373520347	0.013144095	0.999970281
PI16	ENSG00000164530	687.8999627	0.456623688	0.013440402	0.999970281
GREM1	ENSG00000166923	2071.927535	0.456820235	0.013510201	0.999970281
LGR5	ENSG00000139292	557.5491631	0.456811917	0.013527025	0.999970281
AC092296.2	NA	48.34634408	0.40341115	0.013548667	0.999970281
TEKT4	NA	28.05043248	0.371903141	0.013805205	0.999970281
FCGR2A	ENSG00000143226	197.8950621	0.439302391	0.013920445	0.999970281
CENPF	ENSG00000117724	562.006452	0.414595286	0.014035096	0.999970281

PDE7A	ENSG00000205268	2098.097473	0.243787216	0.014193717	0.999970281
PKP4	ENSG00000144283	27996.46369	0.334403559	0.014275872	0.999970281
GIPR	ENSG00000010310	167.5928382	0.452763671	0.014335626	0.999970281
DMRT2	ENSG00000173253	128.7886921	0.438320792	0.01455467	0.999970281
CPXM2	ENSG00000121898	380.84824	0.389483648	0.014560155	0.999970281
MTL5	ENSG00000132749	115.539768	0.413528759	0.014584991	0.999970281
GLRA2	ENSG00000101958	682.7099273	0.332692767	0.014623718	0.999970281
C12orf59	NA	168.1940892	0.398516112	0.014737818	0.999970281
FAM70A	NA	418.5850398	0.339774421	0.014810142	0.999970281
RP11-21A7A.3	NA	9.072833102	0.390105409	0.014911372	0.999970281
MMD	ENSG00000108960	5139.716909	0.433503907	0.015097282	0.999970281
IBSP	ENSG00000029559	24.68826586	0.43289448	0.015290706	0.999970281
GSN-AS1	ENSG00000235865	873.7557598	0.442253538	0.015291488	0.999970281
CD84	ENSG00000066294	489.86526	0.444827824	0.015426934	0.999970281
HHIPL2	ENSG00000143512	28.52409226	0.43686176	0.015445026	0.999970281
GRIK3	ENSG00000163873	8251.878694	0.381133454	0.015734806	0.999970281
PRR11	ENSG00000068489	178.8168435	0.345087558	0.016000051	0.999970281
KANK4	ENSG00000132854	330.3352971	0.426322552	0.016005554	0.999970281
NEUROG2	ENSG00000178403	27.19273266	0.441913448	0.016587499	0.999970281
RCAN3	ENSG00000117602	257.5653685	0.425529238	0.016643753	0.999970281
DMRTA2	ENSG00000142700	32.94856935	0.387903814	0.016790693	0.999970281
C8orf34	ENSG00000165084	609.7438235	0.419907896	0.016823307	0.999970281
CLN8	ENSG00000182372	4674.282895	0.316197552	0.017176786	0.999970281
HOXD-AS1	NA	382.9381994	0.430819055	0.01723373	0.999970281
GREM2	ENSG00000180875	1668.582759	0.291292586	0.01729144	0.999970281
ARHGEF37	ENSG00000183111	3495.09336	0.353516495	0.01759431	0.999970281
RPL7	ENSG00000147604	1089.871539	0.431761322	0.017691857	0.999970281
IGF2BP2	ENSG00000073792	51.49904108	0.43508997	0.017903428	0.999970281
SLITRK1	ENSG00000178235	7978.732406	0.310611819	0.017947953	0.999970281
POM121L2	ENSG00000158553	13.088795	0.366643152	0.017989854	0.999970281
BHMT	ENSG00000145692	130.1717618	0.434587005	0.018378635	0.999970281
COL25A1	ENSG00000188517	453.6132206	0.371871108	0.018501816	0.999970281
DNASE1	ENSG00000213918	784.4462634	0.242103058	0.01856814	0.999970281
UNC5C	ENSG00000182168	4013.511838	0.345324931	0.018681341	0.999970281
LHPP	ENSG00000107902	5684.511378	0.397598987	0.018727057	0.999970281
PIP	ENSG00000159763	12.56977125	0.368216965	0.018764902	0.999970281
IGSF10	ENSG00000152580	199.8578745	0.400824807	0.018830976	0.999970281
XIRP1	ENSG00000168334	16.63763777	0.415061076	0.01895783	0.999970281
CCDC167	ENSG00000198937	770.9787873	0.306194541	0.019085228	0.999970281
NTNG2	ENSG00000196358	4708.288828	0.30663037	0.019285477	0.999970281
snoU2-30	NA	2.584470502	0.285456865	0.019346506	NA
FCGR2C	ENSG00000244682	51.28538274	0.433360825	0.019405195	0.999970281
PCDH9	ENSG00000184226	34578.0301	0.251148267	0.019414595	0.999970281
TMEM97	NA	1505.795303	0.324562002	0.019438761	0.999970281
DIXDC1	NA	7554.21208	0.362941844	0.019450007	0.999970281
NAV3	ENSG00000067798	10630.42627	0.286002836	0.019584016	0.999970281
FILIP1	ENSG00000118407	1412.893991	0.374462636	0.019918342	0.999970281
TBC1D3H	NA	9.440892098	0.248788157	0.019979859	0.999970281

SLITRK6	ENSG00000184564	33.61402717	0.425360907	0.020328305	0.999970281
AL096869.5	NA	66.40469171	0.422793648	0.020950716	0.999970281
HOXD1	ENSG00000128645	130.4948279	0.406977559	0.020977684	0.999970281
MMP2	ENSG00000087245	325.3424402	0.381423198	0.021097289	0.999970281
GRIK1	ENSG00000171189	1867.878538	0.296386875	0.021362212	0.999970281
ZBTB8A	ENSG00000160062	43.95284612	0.425757401	0.021528273	0.999970281
GPD1	ENSG00000167588	1486.686451	0.403507856	0.021766889	0.999970281
CRNDE	ENSG00000245694	20.29118009	0.412761528	0.021774724	0.999970281
TMEM98	ENSG00000006042	2706.066605	0.405075223	0.021846526	0.999970281
H2BFM	NA	3.011646455	0.241502967	0.021895422	0.999970281
SLN	ENSG00000170290	184.7512155	0.421404613	0.021895734	0.999970281
RP11-193H5.1	NA	61.34622328	0.423263117	0.021964714	0.999970281
IRS4	ENSG00000133124	81.97953013	0.407097846	0.022112714	0.999970281
RP11-467L20.9	NA	138.1724021	0.423260147	0.022437351	0.999970281
EPHB2	ENSG00000133216	2177.498258	0.297571592	0.022450009	0.999970281
GRIN3A	ENSG00000198785	2732.068798	0.385100739	0.022521962	0.999970281
SLC27A6	ENSG00000113396	152.4074333	0.313626263	0.022669177	0.999970281
RP3-455J7.4	NA	24.07999165	0.421973761	0.022721406	0.999970281
PCDHB16	NA	370.5267705	0.338112719	0.022744055	0.999970281
COL15A1	ENSG00000204291	185.0754966	0.38307281	0.02289926	0.999970281
SAMSN1	ENSG00000155307	134.112993	0.416308217	0.022941608	0.999970281
FGL2	ENSG00000127951	409.6365257	0.393851511	0.023096245	0.999970281
PRRT3-AS1	ENSG00000230082	51.87743447	0.41416593	0.023443973	0.999970281
TMEM215	ENSG00000188133	87.77520355	0.402658814	0.023736369	0.999970281
CABP5	ENSG00000105507	5.395757084	0.334971137	0.023904235	0.999970281
TMEM156	ENSG00000121895	64.83975039	0.405011399	0.024176854	0.999970281
MBP	ENSG00000197971	633512.4152	0.414570311	0.02430358	0.999970281
KDM5C	NA	11692.15259	0.306035638	0.024475135	0.999970281
TMEM37	ENSG00000171227	45.96915032	0.416475588	0.024498629	0.999970281
NDN	ENSG00000182636	4158.36266	0.252602937	0.024563219	0.999970281
GLT25D2	NA	7616.067556	0.234160402	0.024603975	0.999970281
CTGF	ENSG00000118523	1442.118445	0.337334664	0.024725197	0.999970281
KIR3DX1	ENSG00000104970	16.21393964	0.416282504	0.024725348	0.999970281
KCNJ5	ENSG00000120457	140.8747311	0.403772576	0.024781955	0.999970281
PCDHB8	NA	127.7508599	0.415914161	0.024870589	0.999970281
TRIM67	ENSG00000119283	560.5520734	0.262639161	0.025051602	0.999970281
C5orf64	ENSG00000178722	276.1727136	0.377643205	0.025136377	0.999970281
EDIL3	ENSG00000164176	17005.16571	0.398620652	0.025199506	0.999970281
HLA-DRB5	ENSG00000198502	385.0887332	0.289065476	0.025417994	0.999970281
CKAP2L	ENSG00000169607	37.38356505	0.399746567	0.025500949	0.999970281
CHN2	ENSG00000106069	5008.493275	0.329660731	0.025548145	0.999970281
HLA-P	NA	35.87220681	0.387957521	0.025646842	0.999970281
FAM19A4	ENSG00000163377	231.118089	0.394426109	0.026056388	0.999970281
CETP	ENSG00000087237	62.40174401	0.406708785	0.026058407	0.999970281
C9orf66	ENSG00000183784	16.16615253	0.410645517	0.02616357	0.999970281
CTC-281M20.1	NA	12.15882489	0.40734193	0.026246273	0.999970281
CYP26B1	ENSG00000003137	3958.379538	0.347966566	0.026366057	0.999970281
NOV	ENSG00000136999	2469.440008	0.301819799	0.02636769	0.999970281

AP000569.8	NA	60.76573285	0.390234661	0.026600488	0.999970281
SLC35F1	ENSG00000196376	9916.755073	0.246263257	0.026794369	0.999970281
CCR1	ENSG00000163823	138.6515622	0.407799517	0.026936118	0.999970281
AGA	ENSG00000038002	966.5469856	0.342039646	0.027006192	0.999970281
CTSL2	NA	89.28879803	0.403516644	0.027061091	0.999970281
SIGLEC11	ENSG00000161640	33.54099239	0.395521876	0.027231979	0.999970281
HDHD1	ENSG00000130021	988.2469188	0.310522146	0.027394271	0.999970281
MSH4	ENSG00000057468	136.0080733	0.373238588	0.027479257	0.999970281
DAPK2	ENSG00000035664	1157.35162	0.38072221	0.027527085	0.999970281
HMGCS1	ENSG00000112972	16931.80249	0.320986944	0.0276357	0.999970281
TMEM169	ENSG00000163449	1263.920115	0.301575163	0.027808917	0.999970281
KLRC2	ENSG00000205809	49.187353	0.342259695	0.028207988	0.999970281
SYT6	ENSG00000134207	595.7161677	0.371954505	0.028282202	0.999970281
ADAMTS9-AS1	ENSG00000241158	33.14931726	0.403518039	0.028283267	0.999970281
PDYN	ENSG00000101327	443.0211677	0.403925275	0.028417264	0.999970281
SQLE	ENSG00000104549	3726.489848	0.372960285	0.028459344	0.999970281
AQP7	ENSG00000165269	130.204447	0.403972897	0.028738318	0.999970281
LSM3	ENSG00000170860	2580.52285	0.24092547	0.028815688	0.999970281
AQP12B	ENSG00000185176	12.84929819	0.379987427	0.029069722	0.999970281
RP11-179G5.4	NA	5190.482767	0.328424072	0.029252928	0.999970281
C11orf24	ENSG00000171067	1700.710281	0.2753737	0.029530224	0.999970281
RIMS1	ENSG00000079841	17990.37243	0.27838108	0.029764118	0.999970281
SSH2	ENSG00000141298	3961.584227	0.230040682	0.029791061	0.999970281
PCDH20	ENSG00000197991	2873.7579	0.325805813	0.029903488	0.999970281
KCNK2	ENSG00000082482	1068.357502	0.326598584	0.029974898	0.999970281
CXorf21	ENSG00000120280	45.7466877	0.399759363	0.030134494	0.999970281
ISM1	ENSG00000101230	163.4184749	0.339121569	0.03015423	0.999970281
RP11-545I5.3	NA	353.8197072	0.310410804	0.030372722	0.999970281
CRHBP	ENSG00000145708	1539.669535	0.359291025	0.031036855	0.999970281
RASGRP3	ENSG00000152689	2413.088906	0.383875814	0.031368579	0.999970281
RPL36	ENSG00000130255	12117.42584	0.34131079	0.03155205	0.999970281
TNFSF15	ENSG00000181634	11.22534256	0.394639093	0.031593985	0.999970281
HLA-J	NA	11.73312701	0.370846583	0.031648974	0.999970281
CNTN6	ENSG00000134115	1229.983069	0.3827003	0.031714348	0.999970281
AC022148.1	NA	218.9463694	0.381941141	0.031786494	0.999970281
LIPA	ENSG00000107798	6930.027509	0.375903563	0.031823258	0.999970281
HMSD	ENSG00000221887	90.23849671	0.39442074	0.031943109	0.999970281
RP11-285F7.2	NA	389.2742986	0.395609414	0.032364226	0.999970281
NKAIN2	ENSG00000188580	6348.817251	0.337842129	0.032373442	0.999970281
GUCY1A3	ENSG00000164116	4026.35955	0.305876428	0.032411962	0.999970281
EPHA5	ENSG00000145242	5427.823065	0.351424924	0.032487827	0.999970281
RP5-872K7.7	NA	3.985500932	0.30339687	0.032533003	0.999970281
MKI67	ENSG00000148773	38.50729458	0.383422375	0.032580964	0.999970281
TGFA	NA	1130.089223	0.390328194	0.032770446	0.999970281
F13A1	ENSG00000124491	562.8763945	0.331282756	0.033024233	0.999970281
CEACAM21	NA	33.33706289	0.393550964	0.033334207	0.999970281
CTD-2269F5.1	NA	94.16479981	0.357835716	0.033615872	0.999970281
RP11-536K7.3	NA	10.39950339	0.3560385	0.033809025	0.999970281

VAX1	ENSG00000148704	103.5116951	0.376867764	0.033827355	0.999970281
CT45A5	NA	2.019569092	0.255559312	0.033995374	NA
ROBO2	ENSG00000185008	7409.083563	0.316686776	0.034273884	0.999970281
AC003075.4	NA	11.4800025	0.369887859	0.034336473	0.999970281
RASGRP1	ENSG00000172575	7377.985302	0.338197438	0.034358711	0.999970281
ERAP2	ENSG00000164308	667.2305118	0.392102181	0.034365313	0.999970281
SLC9A2	ENSG00000115616	233.3896507	0.365480703	0.034547884	0.999970281
RP4-788L13.1	NA	13939.8878	0.3414297	0.034561642	0.999970281
GJC2	ENSG00000198835	1006.90193	0.371615431	0.034565878	0.999970281
TRAF3IP3	ENSG00000009790	231.3423317	0.342366957	0.035675725	0.999970281
FGD3	ENSG00000127084	280.285024	0.331624373	0.035962702	0.999970281
MCHR2	ENSG00000152034	2120.96244	0.335465954	0.036170478	0.999970281
ST8SIA2	ENSG00000140557	167.7679199	0.38771355	0.036505045	0.999970281
AC002055.4	NA	1670.814504	0.255512773	0.036527681	0.999970281
MIR25	ENSG00000207547	2.515574342	0.266039231	0.036633854	NA
WNT16	ENSG00000002745	141.9750752	0.323497042	0.036694139	0.999970281
CYP1A1	ENSG00000140465	114.3867413	0.362337687	0.036756745	0.999970281
ERMN	ENSG00000136541	25666.64865	0.386769825	0.036972087	0.999970281
AC079135.1	NA	7.08515741	0.333736171	0.037069627	0.999970281
HTR2C	NA	694.8344618	0.329249469	0.037109507	0.999970281
FAM5C	NA	2248.128573	0.256176679	0.037244194	0.999970281
RP1-163M9.6	NA	36.18959581	0.384248064	0.037333724	0.999970281
NMU	ENSG00000109255	43.87128491	0.385399904	0.037640454	0.999970281
MPEG1	ENSG00000197629	224.2188352	0.38044311	0.037666328	0.999970281
AIF1	NA	420.2525645	0.380828018	0.038080937	0.999970281
RAB3C	ENSG00000152932	31980.07718	0.326949952	0.038223033	0.999970281
LRP2	ENSG00000081479	3832.902931	0.383795132	0.038375494	0.999970281
ACCSL	ENSG00000205126	3.88912116	0.287429484	0.038514911	0.999970281
RGPD5	NA	246.3905301	0.382963564	0.038765952	0.999970281
RP11-111E14.1	NA	96.8222492	0.374020471	0.039174577	0.999970281
TSPAN11	ENSG00000110900	694.6881622	0.341559814	0.039385934	0.999970281
EPM2A	ENSG00000112425	1657.36637	0.214297118	0.039408479	0.999970281
C2orf89	NA	1542.29035	0.306400995	0.039481855	0.999970281
PLEK	ENSG00000115956	218.3481865	0.353330776	0.039513678	0.999970281
BFSP1	ENSG00000125864	367.1978505	0.27875024	0.039967323	0.999970281
C1QA	ENSG00000173372	619.3769413	0.3804454	0.0400365	0.999970281
SYNJ2-IT1	ENSG00000233496	10.17928215	0.369288894	0.040279996	0.999970281
ANLN	ENSG00000011426	9059.705508	0.379046704	0.040300765	0.999970281
PELI1	ENSG00000197329	1756.499633	0.284565601	0.040304448	0.999970281
PHF16	NA	558.3845014	0.338953659	0.040365243	0.999970281
IL21R	ENSG00000103522	118.8808944	0.372122371	0.040498209	0.999970281
PLP1	ENSG00000123560	259744.6508	0.379027058	0.040585709	0.999970281
SIGLEC10	ENSG00000142512	166.5824465	0.378219095	0.040634674	0.999970281
CCR6	NA	144.1019168	0.276500751	0.040680827	0.999970281
ZSCAN29	ENSG00000140265	2477.04515	0.230104647	0.040807506	0.999970281
ASAH1	ENSG00000104763	9323.558292	0.186474892	0.040834943	0.999970281
LANCL1	ENSG00000115365	29038.42095	0.29070757	0.041004769	0.999970281
GABRA5	ENSG00000186297	10371.86127	0.291814612	0.041314108	0.999970281

ODZ3	NA	7168.051385	0.295809866	0.041326806	0.999970281
PLAC2	NA	807.0586095	0.329369004	0.041377789	0.999970281
ZMIZ1	ENSG00000108175	13284.71642	0.197870847	0.041480758	0.999970281
SDK2	ENSG00000069188	2463.095507	0.253830905	0.041504871	0.999970281
TMEM199	NA	1137.366317	0.196292068	0.041531315	0.999970281
INSIG1	ENSG00000186480	4770.216435	0.322716755	0.041551687	0.999970281
C1orf95	ENSG00000203685	19657.62644	0.340770259	0.041641246	0.999970281
LINC00471	ENSG00000181798	144.3341778	0.362213115	0.041662686	0.999970281
BCHE	ENSG00000114200	502.445992	0.368769956	0.04176271	0.999970281
AC092667.2	NA	61.32109046	0.372018964	0.042010919	0.999970281
C8orf51	NA	57.67528725	0.332595517	0.042346846	0.999970281
LDB3	ENSG00000122367	3460.540872	0.365707944	0.042367496	0.999970281
ZNF595	ENSG00000272602	249.4878432	0.370986539	0.042501826	0.999970281
CEACAM7	NA	2.464892448	0.207711567	0.042552336	NA
SPRN	ENSG00000203772	4117.227723	0.297515244	0.042761923	0.999970281
C6orf141	ENSG00000197261	182.4310378	0.374898025	0.042837024	0.999970281
GCNT1	ENSG00000187210	680.5751148	0.292343192	0.042946697	0.999970281
FGF1	ENSG00000113578	14239.0475	0.333591202	0.042971044	0.999970281
SLC25A13	ENSG00000004864	1073.026286	0.347477074	0.043209682	0.999970281
TFRC	ENSG00000072274	7247.918297	0.344423674	0.043219346	0.999970281
COX10-AS1	ENSG00000236088	385.7919653	0.228651228	0.043264559	0.999970281
TNFSF14	ENSG00000125735	6.242554276	0.326962953	0.043423167	0.999970281
LANCL2	ENSG00000132434	8258.321063	0.224571241	0.043522325	0.999970281
GALNTL6	ENSG00000174473	801.8287348	0.30350985	0.043607793	0.999970281
RP5-1043L13.1	NA	94.69202743	0.372512569	0.043644196	0.999970281
HCG4P3	NA	16.71254646	0.346014491	0.043710949	0.999970281
TUBA3E	ENSG00000152086	18.92243319	0.36056947	0.043833944	0.999970281
C15orf37	NA	53.49249619	0.316618369	0.044426245	0.999970281
ADAMTS12	ENSG00000151388	41.45747855	0.353410607	0.044474333	0.999970281
RP11-959I15.3	NA	45.30096989	0.359506691	0.04455038	0.999970281
SLC24A1	ENSG00000074621	958.8777129	0.157278769	0.044569604	0.999970281
ZEB2-AS1	ENSG00000238057	11.86829852	0.352627236	0.044586231	0.999970281
NPPA	ENSG00000175206	133.916615	0.362427828	0.044815205	0.999970281
LYVE1	ENSG00000133800	337.490123	0.344462086	0.044908153	0.999970281
OLR1	ENSG00000173391	517.310571	0.360695775	0.044958616	0.999970281
SEMA3E	ENSG00000170381	1821.537633	0.356129623	0.045032577	0.999970281
POC1A	ENSG00000164087	287.0389294	0.318402898	0.045180971	0.999970281
MXRA5	ENSG00000101825	255.553269	0.367040389	0.04551957	0.999970281
TMEM200A	ENSG00000164484	801.0070607	0.32876024	0.045677079	0.999970281
AC010894.5	NA	6.323511611	0.336058073	0.045880995	0.999970281
SOX2-OT	ENSG00000242808	15882.31463	0.369611683	0.045981992	0.999970281
WNK1	ENSG00000060237	42238.86004	0.260561655	0.046114168	0.999970281
FUT9	ENSG00000172461	448.2951848	0.369614754	0.04618895	0.999970281
TLL1	ENSG00000038295	303.5099765	0.266302624	0.046494467	0.999970281
TFEC	ENSG00000105967	93.37121206	0.368072701	0.04710687	0.999970281
BVES	ENSG00000112276	685.829484	0.3388693	0.047412399	0.999970281
PHGR1	ENSG00000233041	1.78193764	0.237896932	0.047508906	NA
RGS10	ENSG00000148908	382.3929018	0.364424218	0.047581371	0.999970281

NLGN4X	ENSG00000146938	5818.581161	0.304831281	0.047837674	0.999970281
NKX6-1	ENSG00000163623	21.59863819	0.366406895	0.047870755	0.999970281
SFRP1	ENSG00000104332	1724.768893	0.330870096	0.047984475	0.999970281
ABCA8	ENSG00000141338	4300.716462	0.366629077	0.047991089	0.999970281
CCDC109B	ENSG00000005059	256.8957128	0.314674721	0.048073424	0.999970281
KRT6C	ENSG00000170465	1.688316378	0.155329505	0.048187667	NA
TRIM59	ENSG00000213186	626.3486331	0.364751548	0.048247007	0.999970281
NINJ2	ENSG00000171840	528.6345046	0.359059814	0.048471019	0.999970281
ZNF26	NA	2.96936002	0.203586553	0.04857669	0.999970281
EPHA7	ENSG00000135333	1964.091827	0.262716314	0.04870352	0.999970281
SGMS2	ENSG00000164023	583.9365667	0.317478603	0.048800196	0.999970281
NPY	ENSG00000122585	2064.804873	0.359956983	0.048809022	0.999970281
PCDHAC1	NA	405.8505941	0.305896204	0.048848782	0.999970281
KCNQ1OT1	ENSG00000269821	2481.587161	0.334207502	0.049334452	0.999970281
SLC35D2	ENSG00000130958	636.2619788	0.356494043	0.049384007	0.999970281
TP53INP2	ENSG00000078804	21244.73976	0.343661961	0.049391877	0.999970281
BACE1	ENSG00000186318	16277.2865	0.254363961	0.049559256	0.999970281
